# Lung-specific MCEMP1 functions as an adaptor for KIT to promote SCF-mediated mast cell proliferation

**DOI:** 10.1038/s41467-023-37873-3

**Published:** 2023-04-11

**Authors:** Youn Jung Choi, Ji-Seung Yoo, Kyle Jung, Logan Rice, Dokyun Kim, Violetta Zlojutro, Matthew Frimel, Evan Madden, Un Yung Choi, Suan-Sin Foo, Younho Choi, Zhongyi Jiang, Holly Johnson, Mi-Jeong Kwak, Seokmin Kang, Brian Hong, Gil Ju Seo, Stephanie Kim, Shin-Ae Lee, Samad Amini-Bavil-Olyaee, Hadi Maazi, Omid Akbari, Kewal Asosingh, Jae U. Jung

**Affiliations:** 1 Department of Cancer Biology, Infection Biology Program, and Global Center for Pathogen and Human Health Research, Lerner Research Institute, Cleveland Clinic, Cleveland, OH 44195 USA; 2 Department of Molecular Microbiology and Immunology, Keck School of Medicine, University of Southern California, Los Angeles, CA 90033 USA; 3 School of Life Sciences, BK21 FOUR KNU Creative BioResearch Group, Kyungpook National University, Daegu, 41566 South Korea; 4 Department of Inflammation and Immunity, Lerner Research Institute, Cleveland Clinic, Cleveland, OH 44195 USA; 5 Florida Research and Innovation Center, Cleveland Clinic, Port Saint Lucie, FL 34987 USA; 6 Biosafety Development Group, Cellular Sciences Department, Amgen Inc., One Amgen Center Drive, Thousand Oaks, CA 91320 USA

**Keywords:** Asthma, Chronic inflammation, Mechanisms of disease, Mast cells, Mucosal immunology

## Abstract

Lung mast cells are important in host defense, and excessive proliferation or activation of these cells can cause chronic inflammatory disorders like asthma. Two parallel pathways induced by KIT–stem cell factor (SCF) and FcεRI–immunoglobulin E interactions are critical for the proliferation and activation of mast cells, respectively. Here, we report that mast cell-expressed membrane protein1 (MCEMP1), a lung-specific surface protein, functions as an adaptor for KIT, which promotes SCF-mediated mast cell proliferation. MCEMP1 elicits intracellular signaling through its cytoplasmic immunoreceptor tyrosine-based activation motif and forms a complex with KIT to enhance its autophosphorylation and activation. Consequently, MCEMP1 deficiency impairs SCF-induced peritoneal mast cell proliferation in vitro and lung mast cell expansion in vivo. *Mcemp1*-deficient mice exhibit reduced airway inflammation and lung impairment in chronic asthma mouse models. This study shows lung-specific MCEMP1 as an adaptor for KIT to facilitate SCF-mediated mast cell proliferation.

## Abstract (web summary)

Mast cells are activated and proliferate during allergic reactions which can involve mast cell specific proteins. Here the authors show that mast cell-expressed membrane protein1 (MCEMP1) is an adaptor for KIT to promote SCF mediated mast cell proliferation and lack of MCEMP1 reduces inflammation in mouse asthma models.

## Introduction

Mast cell-expressed membrane protein1 (MCEMP1) is a type II transmembrane protein that is highly expressed in human lung tissue^1^. MCEMP1 is primarily expressed in myeloid lineage immune cells, such as lung-resident mast cells and alveolar macrophages. MCEMP1 is one of the top inducible genes in many inflammatory diseases, such as asthma^2–4^, idiopathic pulmonary fibrosis^5,6^, cancer^7^, sepsis^8,9^, and stroke^10,11^. Mounting evidence suggests MCEMP1 as a critical factor in allergic and inflammatory lung diseases, with the level of MCEMP1 expression positively correlates with disease severity. For instance, asthma patients at exacerbation stage exhibit significantly higher expression of MCEMP1 compared to the recovery stage^2^. Furthermore, a single cell mass cytometry analysis of patients at high risk of idiopathic pulmonary fibrosis shows higher expression of MCEMP1 in granulocytes than those from patients at low risk^6^. Collectively, these findings indicate that MCEMP1 may have a crucial function in pulmonary inflammation. However, the underlying molecular mechanism by which MCEMP1 contributes to the pathogenesis of lung inflammatory diseases has not been understood.

Mast cells are primarily located on tissues that are exposed to the external environment such as skin, conjunctiva, lung, and gastrointestinal track, as well as other tissues such as brain meninges and adipose tissue^12,13^. Although rare in population, mast cells indispensably contribute a unique and versatile function in human health and disease. As one of the important tissue-resident immune cells, mast cells elicit host defense immune responses against helminths infection^14,15^. Mast cells also provide protection against bee and snake venoms, as well as endogenous peptide toxins such as endothelin-1 and neurotensin, by producing peptidases to inactivate the toxins^16–20^. On the other hand, aberrant proliferation or activation of mast cells is associated not only with many allergic disorders such as asthma, atopic dermatitis, systemic anaphylaxis, mast cell activation syndrome, and mastocytosis, but also with nonallergic conditions, including stroke and cardiovascular diseases^21–24^.

Mast cells respond to various external signals through the coordination of numerous activating and inhibitory surface receptors, including high-affinity IgE receptor (FcεRI) and KIT receptor^25^. FcεRI activates mast cells, while the KIT receptor promotes mast cell proliferation. When exposed to allergens, the high-affinity receptor FcεRI is crosslinked by IgE, leading to rapid degranulation of mast cells and the release of various granule-stored mediators, including bioactive amines (histamine and serotonin), mast cell proteases (tryptase, chymase, and carboxypeptidase), proteoglycans (heparin), β-hexosaminidase, lipid mediators (leukotrienes and prostaglandins), neuropeptide substance P, and other cytokines and chemokines^26^. Subsequently, mast cell mediators recruit immune cells, increase vascular permeability and smooth muscle contraction, and activate epithelial, neuronal, and stromal cells^27,28^.

On the other hand, KIT is a type III receptor tyrosine kinase that provides signals for the growth and survival of mast cells. Activation of KIT occurs upon binding to either the membrane-bound or soluble form of stem cell factor (SCF), a growth factor that is expressed by endothelial cells and fibroblasts. SCF-mediated activation of KIT leads to autophosphorylation of its own tyrosine residues in the intracellular region, acting as docking sites for downstream signaling molecules^29^. Structural analysis shows that binding of SCF induces dimerization or oligomerization of KIT, which leads to trans-autophosphorylation of KIT and the subsequent release of its autoinhibitory constraint^30–33^. Activation of KIT induces downstream signal transduction pathways, including mitogen-activated protein kinase (MAPK) pathway, phosphatidylinositol 3´-kinase (PI3K)-AKT pathway, phospholipase C pathway, and Lyn and Lck tyrosine kinase signaling pathway. The signaling amplitude of SCF-KIT axis in mast cells is dependent on full KIT receptor agonism, thus engineered SCF that acts as a partial agonist impedes KIT dimerization and fails to induce mast cell proliferation^34^.

The essential activity of KIT in mast cell proliferation is demonstrated in mice with *Kit* mutations, such as *Kit*^w-sh^ and *Kit*^w-v^, which exhibit mast cell-deficiency and have been commonly used to investigate the role of mast cells in allergic disorders^35,36^. Also, aberrant KIT kinase activity has been linked to various cancers and allergic disorders^29^. For instance, the oncogenic KIT mutations, such as KIT D816V and KIT V560G, lead to constitutively active tyrosine kinase activity and growth factor-independent proliferation of mast cells in tissue lesions associated with mast cell tumors and systemic mastocytosis^37,38^. Thus, multi-selective kinase inhibitors such as imatinib and dasatinib have been used clinically to reduce mast cell burdens in cancer and other allergic disorders^39–41^.

Particularly, the SCF-KIT signaling is engaged in maintaining mast cells within lung tissue. The extent of mast cells accumulation in inflamed areas of allergic lung tissues correlates with the severity and progression of asthma, underscoring the pivotal function of mast cells in the pathophysiology of asthma^35,36,42,43^. However, despite this striking association, it remains unclear how mast cells expand within the lungs of individuals with asthma and whether dysregulation of mast cell proliferation influence the progression and severity of asthma.

In this work, we elucidate the pathological activity of MCEMP1-mediated amplification of KIT receptor signaling in mast cell proliferation, and its impact on the progression of airway inflammation and lung function impairment in a chronic asthma mouse model. This discovery of MCEMP1-mediated mast cell expansion could be important to the development of therapeutic strategies to alleviate airway mast cell burdens in severe or poorly controlled lung inflammation.

## Results

### MCEMP1 induces intracellular signal transduction in an ITAM-dependent manner

**Fig. 1:**
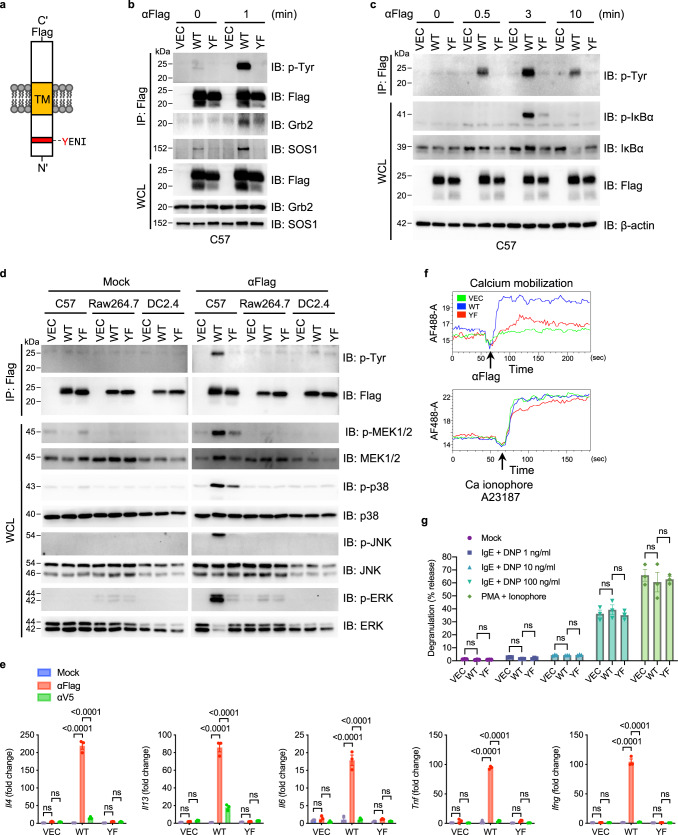
MCEMP1 has an ITAM-dependent signal-transducing activity in mast cell. **a** Schematic diagram of MCEMP1 structure. ITAM motif, YENI; TM, transmembrane. **b** MCEMP1 tyrosine phosphorylation and its interaction with Grb2 and SOS1. C57 cells expressing vector (VEC), wild-type MCEMP1 (WT), or tyrosine to phenylalanine mutant MCEMP1 (YF) were treated with αFlag for 1 min and cell lysates were immunoprecipitated (IP) with anti-Flag antibody. Immunoprecipitates and whole cell lysates (WCL) were analyzed by immunoblotting (IB) with the indicated antibodies. The upper band of MCEMP1 is a glycosylated form. **c** IP and IB analysis of MCEMP1 phosphorylation and downstream NF-κB signaling in C57 cells. **d** Mast cell-specific activation of MCEMP1 and downstream MAPK signal transduction in C57, Raw264.7, or DC2.4 cells. **e** Gene expression of *Il4*, *Il13*, *Il6*, *Tnf*, and *Ifng* in C57 cells after αFlag treatment. Mock or αV5 antibody was treated as negative controls. **f** Intracellular calcium influx upon αFlag-mediated MCEMP1 activation. Calcium Ionophore was used as a positive control. **g** β-hexosaminidase assay measuring mast cell degranulation. PMA + ionophore (positive control); dinitrophenyl (DNP) + α-DNP-IgE (FcεRI activation). Data are representative of at least two independent experiments in **b**–**f**. Data are presented by mean±s.e.m. and *p*-values were determined by two-way ANOVA with Tukey’s comparison in **g** (*n* = 3) and **e** (*n* = 3). ns, not significant.

The cytoplasmic domain of MCEMP1 contains a highly conserved immunoreceptor tyrosine-based activation motif (ITAM), an essential signaling motif that links immunoreceptors to downstream signaling machinery (Fig. 1a and Supplementary Fig. 1a). To investigate the functional phosphorylation of the putative cytoplasmic ITAM, we substituted the tyrosine residue with phenylalanine (referred to as YF). In addition, we incorporated a Flag epitope at the carboxyl-terminal extracellular domain of MCEMP1 to employ anti-Flag antibody-mediated stimulation of MCEMP1. When C57 mouse mast cells or HMC-1 human mast cells stably expressing either wild-type (WT) or mutant (YF) MCEMP1 were stimulated with Flag antibody, WT MCEMP1, but not the YF mutant, was rapidly phosphorylated (Fig. 1b and Supplementary Fig. 1f).

To identify cellular proteins interacting with the cytoplasmic ITAM-containing domain of MCEMP1, a GST pull-down assay was performed using bacterial GST fusion proteins. Tyrosine-phosphorylated GST/MCEMP1-C(P) was produced from *E. coli* strain *TKX1*, which contains the elk tyrosine kinase. Wild-type GST/MCEMP1-C(P) fusion protein (GST-WT) or mutant protein (GST-YF) purified from *TKX1* was used as an affinity column for HMC-1 cell lysates (Supplementary Fig. 1b). In addition, lysates of C57 cells expressing MCEMP1 WT were subjected to protein purification with anti-Flag antibody. Mass spectrometry analysis of polypeptides that specifically interacted with GST-WT or full-length MCEMP1 WT, but not with GST or GST-YF mutant, identified two adaptor signaling proteins, proline-rich-motif-containing son of sevenless 1/2 (SOS1/2) and SH2-SH3 domain-containing growth factor receptor-bound protein 2 (Grb2) (Supplementary Fig. 1b,c and Supplementary Table 1). When lysates of 293T cells exogenously expressing SOS1 or SOS2 were used for GST pull-down assay, GST-WT readily bound to endogenous Grb2 and exogenous SOS1/2 (Supplementary Fig. 1d). In contrast, GST and GST-YF showed no detectable interaction with Grb2 or SOS1/2. Interestingly, treatment of the SOS SH3 domain inhibitor (VPPPVPPRRR), a biologically active peptide that blocks SOS-Grb2 interaction, effectively reduced SOS-GST-WT interaction without affecting Grb2-GST-WT interaction (Supplementary Fig. 1e). Finally, full-length MCEMP1 readily interacted with Grb2 and SOS1 in C57 or HMC-1 cells in an ITAM-dependent manner (Fig. 1b and Supplementary Fig. 1f). These results suggest that the SH2 domain of Grb2 interacts with the cytoplasmic ITAM of MCEMP1 in a tyrosine phosphorylation-dependent manner, whereas the SH3 domain of Grb2 interacts with the proline-rich motif of SOS1/2, forming a MCEMP1-Grb2-SOS1/2 signalosome.

Stimulation of Flag antibody that induced the activation and phosphorylation of MCEMP1 triggered downstream signal transduction of the nuclear factor kappa-light-chain-enhancer of activated B cells (NF-κB) and MAP kinase pathways (Figs. 1c, d). Strikingly, MCEMP1-mediated activation of MAP kinase pathway was only observed in C57 mast cells, but not in Raw264.7 macrophages or DC2.4 dendritic cells (Fig. 1d). In addition, MCEMP1 activation robustly induced expressions of several cytokine and chemokine genes including *Il4*, *Il13*, *Il6*, *Tnf*, *Ifng*, *Ccl4*, and *Ccl11* in C57 mast cells, but not in DC2.4 dendritic cells or J774.1 macrophages (Fig. 1e and Supplementary Fig. 1g). Flag antibody mediated MCEMP1 activation also induced intracellular calcium mobilization in C57 mast cells (Fig. 1f). On the other hand, MCEMP1 activation showed no effect on DNP-specific IgE-mediated mast cell degranulation, suggesting that MCEMP1 signaling is independent of the FcεRI-IgE pathway (Fig. 1g). Collectively, these data demonstrate that MCEMP1 forms a signalosome with Grb2 and SOS1/2 in a cytoplasmic ITAM-dependent manner, which induces multiple downstream signal transduction and elicits intracellular calcium mobilization and inflammatory cytokine production.

### MCEMP1 enhances SCF-induced mast cell proliferation

**Fig. 2:**
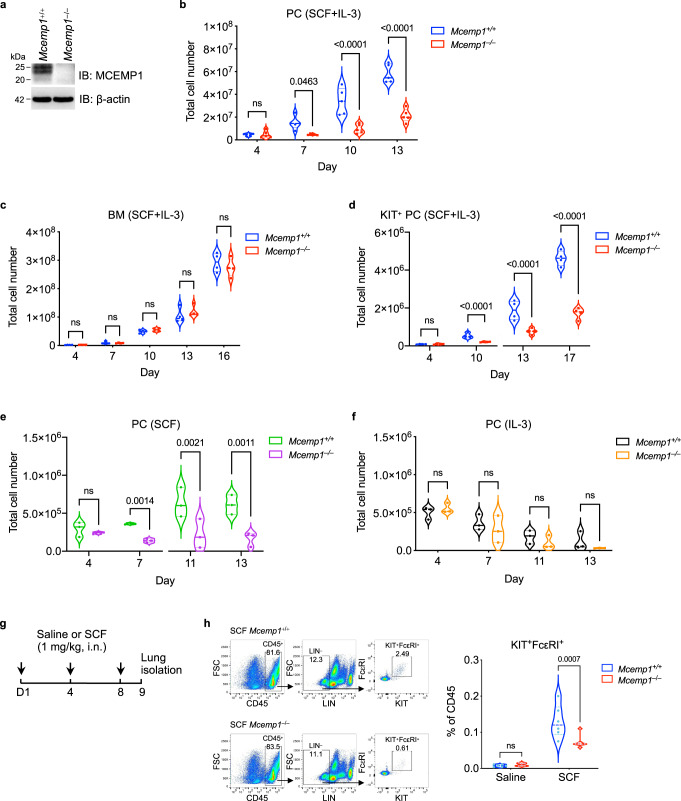
MCEMP1 deficiency impairs SCF-induced mast cell proliferation. **a** Loss of MCEMP1 protein expression in *Mcemp1*^–/–^ mice. Lungs were immunoblotted with MCEMP1 antibody. Blots are representative of two independent experiments. **b**, **c** Cell growth kinetics of *Mcemp1*^+/+^ and *Mcemp1*^–/–^ peritoneal cell (PC, *n* = 5 per group) or bone marrow cells (BM, *n* = 4 per group) cultured with SCF and IL-3 for the indicated days. **d** Cell growth kinetics of *Mcemp1*^+/+^ and *Mcemp1*^–/–^ KIT-positive PC isolated by magnetic beads and cultured with SCF and IL-3 for the indicated days. **e**, **f** Absolute counts of *Mcemp1*^+/+^ and *Mcemp1*^–/–^ PC cultured with either SCF or IL-3 for the indicated days (*n* = 3 per group). **g** Schematics of SCF intranasal (i.n.) challenge of *Mcemp1*^+/+^ and *Mcemp1*^–/–^ mice. **h** Representative flow cytometry plots illustrating the gating strategy to identify KIT/FcεRI double-positive mast cells; CD45^+^LIN^–^KIT^+^FcεRI^+^. The percentages of KIT^+^FcεRI^+^ mast cells in the lungs of *Mcemp1*^+/+^ or *Mcemp1*^–/–^ mice challenged with saline or SCF (*n* = 6-7 mice per group). Data are presented as violin plot with lines at median and quartiles and *p*-values were determined by two-way ANOVA with Sidak’s multiple comparison in **b**, **c**, **d**, **e**, **f**, **h**. ns, not significant.

To investigate a physiological function of MCEMP1 in vivo, we generated *Mcemp1*^–/–^ mice using the CRISPR/Cas9 system. Deletion of 11 nucleotides at the exon 1 of *Mcemp1* resulted in a frameshift that created a premature termination codon (Supplementary Fig. 2a,b). Loss of MCEMP1 expression was confirmed by immunoblotting and immunohistochemistry of the lungs of *Mcemp1*^–/–^ mice (Fig. 2a and Supplementary 2c). To induce primary mast cell differentiation and proliferation in vitro, purified cells from the peritoneal lavage or bone marrow of *Mcemp1*^+/+^ or *Mcemp1*^–/–^ mice were cultured with SCF and IL-3. Strikingly, the numbers of expanded peritoneal cells (PC) from *Mcemp1*^–/–^ mice were markedly lower than those from *Mcemp1*^+/+^ mice (Fig. 2b). In contrast, bone marrow cells (BM) from *Mcemp1*^–/–^ mice showed comparable levels of the numbers to those from *Mcemp1*^+/+^ mice (Fig. 2c). When KIT-positive cells were isolated from the peritoneum lavage using CD117 MicroBeads and subsequently cultured with SCF and IL-3, the numbers of KIT-positive cells from *Mcemp1*^–/–^ mice were also markedly lower than those from *Mcemp1*^+/+^ mice (Fig. 2d). In contrast, no significant differences were observed in the total numbers of bone marrow-derived macrophage from *Mcemp1*^–/–^ mice compared to those from *Mcemp1*^+/+^ mice (Supplementary Fig. 2d). Furthermore, flow cytometry analysis showed that the percentages of KIT^+^FcεRI^+^ PC and BM from *Mcemp1*^–/–^ mice were similar to those from *Mcemp1*^+/+^ mice (Supplementary Fig. 2e, f).

We further examined the effect of individual growth factor, SCF or IL-3, on the proliferation of PC and BM from *Mcemp1*^–/–^ mice or *Mcemp1*^+/+^ mice. It should be noted that SCF or IL-3 individual treatment of PC or BM-induced mast cell proliferation at considerably reduced level than both treatments. Intriguingly, SCF-treated PC from *Mcemp1*^–/–^ mice showed minimal proliferation compared to SCF-treated PC from *Mcemp1*^+/+^ mice, whereas IL-3-induced growth of PC from either *Mcemp1*^–/–^ or *Mcemp1*^+/+^ mice was similar (Figs. 2e, f). In contrast, SCF-treated BM or IL-3-treated BM from *Mcemp1*^–/–^ or *Mcemp1*^+/+^ mice showed minimal or no growth in vitro (Supplementary Fig. 2g, h). This indicates that MCEMP1 enhances SCF-induced mast cell proliferation in vitro. We also performed RNA-seq analysis to investigate whether peritoneal cell-derived mast cells (PCMC) exhibited similar gene expression patterns to pulmonary mast cells. Peritoneal cells were in vitro cultured with SCF and IL-3 for 13 days. KIT and FcεRI double-positive cells (KIT^+^FcεRI^+^) and KIT single-positive cells (KIT^+^FcεRI^–^) were isolated by cell sorter and subjected to RNA-seq (Supplementary Fig. 2i). We utilized the Tabula Muris repository of single-cell transcriptome data as a reference for lung mast cell gene expression^44^. We evaluated single-cell transcriptome of mouse lungs and compiled a list of genes that were specifically upregulated in lung mast cell. The analyses showed that KIT^+^FcεRI^+^ PCMC expressed mast cell signature genes that were similar to those enriched in pulmonary mast cells, including mast cell carboxypeptidase A3 (*Cpa3*), mast cell protease (*Mcpt8*), and specific transcription factor (*Gata2*). These results indicate that the gene expression profiles of peritoneal cell-derived mast cells and pulmonary mast cells are highly similar.

To further address whether MCEMP1 deficiency has an impact on SCF-induced mast cell expansion in vivo, 12-week-old *Mcemp1*^+/+^ or *Mcemp1*^–/–^ mice were intranasally challenged with 1 mg/kg of endotoxin-free SCF or saline every 4 days for a total of 3 times (Fig. 2g). Intranasal instillation of SCF significantly elevated KIT^+^FcεRI^+^ mast cell population among the total CD45^+^ immune cells in the lungs of *Mcemp1*^+/+^ mice (Fig. 2h). In contrast, in vivo SCF-induced lung mast cell expansion was considerably reduced in *Mcemp1*^−/−^ mice compared to *Mcemp1*^+/+^ mice (Fig. 2h). Collectively, these results indicate that MCEMP1 is a critical factor for efficient SCF-induced proliferation of peritoneal mast cells in vitro and lung resident mast cells in vivo.

### MCEMP1 interacts with and activates KIT signaling activity

**Fig. 3:**
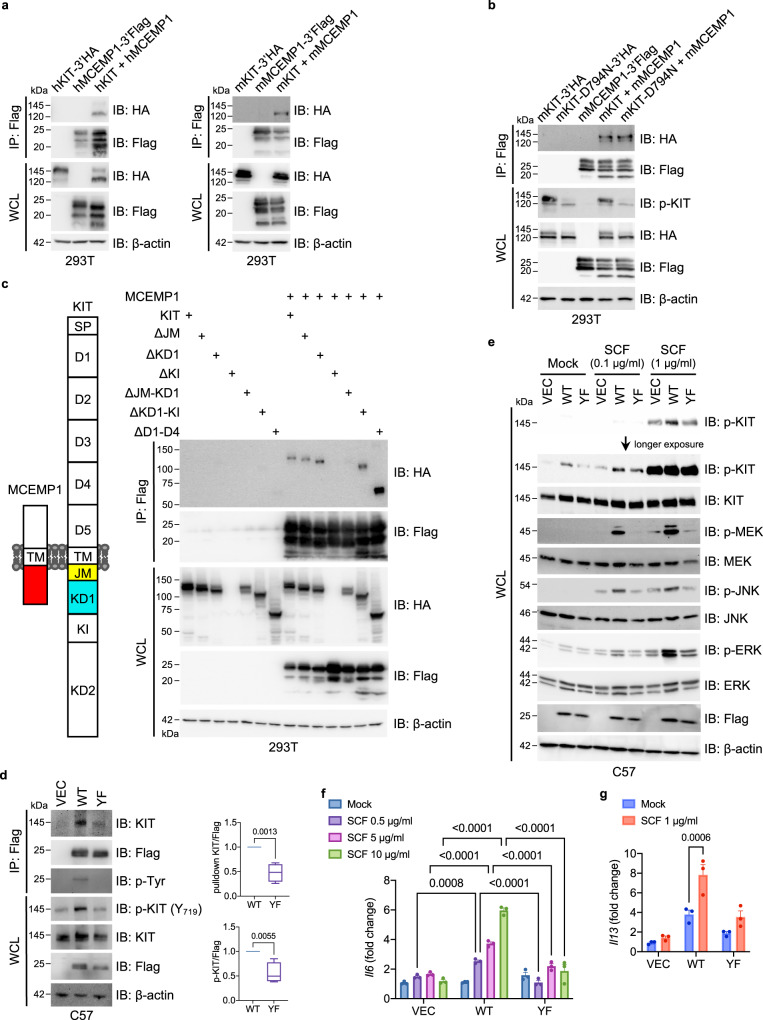
MCEMP1-KIT interaction amplifies downstream signal transduction. **a** Co-immunoprecipitation assay of human and mouse MCEMP1 and KIT interaction in 293 T cells. **b** Co-immunoprecipitation assay of MCEMP1 interaction with wild-type KIT or enzymatic dead (D794N) mutant in 293 T cells. **c** Schematic diagram of MCEMP1 and KIT structure and co-immunoprecipitation assay of MCEMP1 interaction with KIT deletion mutants in 293T cells. ΔKI was not expressed. **d** Immunoprecipitation and Immunoblot analysis of MCEMP1 and KIT phosphorylation in C57 cells expressing vector (VEC), wild-type MCEMP1 (WT), or YF mutant MCEMP1 (YF). C57 cell lysates were immunoprecipitated with anti-Flag antibody. Immunoprecipitates and WCL were analyzed by IB with the indicated antibodies. Band intensity of Immunoprecipitated KIT and phosphorylated KIT was measured with densitometric analysis by ImageJ and normalized to the intensity of MCEMP1 WT or YF. Data are presented by min to max of box and whiskers and *p*-values were determined by two-tailed unpaired Student’s *t*-test (*n* = 4). **e** KIT phosphorylation and downstream MAPK signal transduction upon SCF stimulation in C57 cells expressing VEC, WT MCEMP1 or YF mutant MCEMP1. **f**, **g** Gene expression of *Il6* and *Il13* after SCF stimulation in C57 cells expressing VEC, WT MCEMP1 or YF mutant MCEMP1. Data are representative of at least two independent experiments in **a**–**e**. Data are presented by mean±s.e.m. and *p*-values were determined by two-way ANOVA with Sidak’s multiple comparison in **f** (*n* = 3) and **g** (*n* = 3).

Mechanistically, immunoprecipitation assay demonstrated a strong interaction between human MCEMP1 and human KIT, as well as between mouse MCEMP1 and mouse KIT (Fig. 3a and Supplementary Fig. 3a). We also observed that human MCEMP1 interacted with mouse KIT receptor (Supplementary Fig. 3b). MCEMP1 also interacted with endogenous KIT in C57 mast cells stably expressing MCEMP1 (Supplementary Fig. 3c). KIT kinase-dead mutant (D794N) was capable of binding to MCEMP1, indicating that the kinase activity of KIT is not required for MCEMP1 interaction (Fig. 3b). We further constructed various deletion mutants of KIT to identify the binding domain(s) that are responsible for MCEMP1 binding. The extracellular region of KIT comprises of five Ig-like domains (designated as D1 to D5) and the intracellular region of KIT contains a juxta membrane domain (JM), tyrosine kinase domain-1 (KD1), kinase insert domain (KI), and tyrosine kinase domain-2 (KD2). The extracellular domain deletion mutant (ΔD1-D4) of KIT showed efficient MCEMP1 binding, indicating that the intracellular domain of KIT is critical for the interaction with MCEMP1 (Fig. 3c). While ΔJM or ΔKD1 mutant maintained MCEMP1 binding, ΔJM-KD1 mutant lost MCEMP1 binding, suggesting that either the JM or KD1 domain of KIT is responsible for the binding of MCEMP1 (Fig. 3c). Moreover, the JM and KD1 region (JM-KD1) of KIT efficiently interacted with MCEMP1, indicating that the JM-KD1 region of KIT is sufficient for MCEMP1 interaction (Supplementary Fig. 3d).

Interestingly, ectopic expression of WT MCEMP1, but not YF mutant, increased KIT Y_719_ phosphorylation (Fig. 3d); anti-Flag-mediated MCEMP1 activation further increased KIT phosphorylation (Supplementary Fig. 3e); and expression of WT MCEMP1, but not YF mutant, markedly elevated SCF-mediated KIT phosphorylation and downstream signal transduction including MEK, JNK, and ERK phosphorylation (Fig. 3e). In addition, expression of WT MCEMP1, but not YF mutant, drastically enhanced SCF-induced *Il6* and *Il13* expressions (Figs. 3f, g). In addition, MCEMP1 WT or YF mutant was present as a dimer or trimer form regardless of αFlag or SCF stimulation, indicating that MCEMP1 may function as an oligomer to amplify its signaling activity (Supplementary Fig. 3f). Collectively, these results indicate that MCEMP1 interacts with and activates KIT activity and downstream signal transduction in an ITAM-dependent manner.

### MCEMP1 deficiency alters gene expression related to mast cell proliferation

**Fig. 4:**
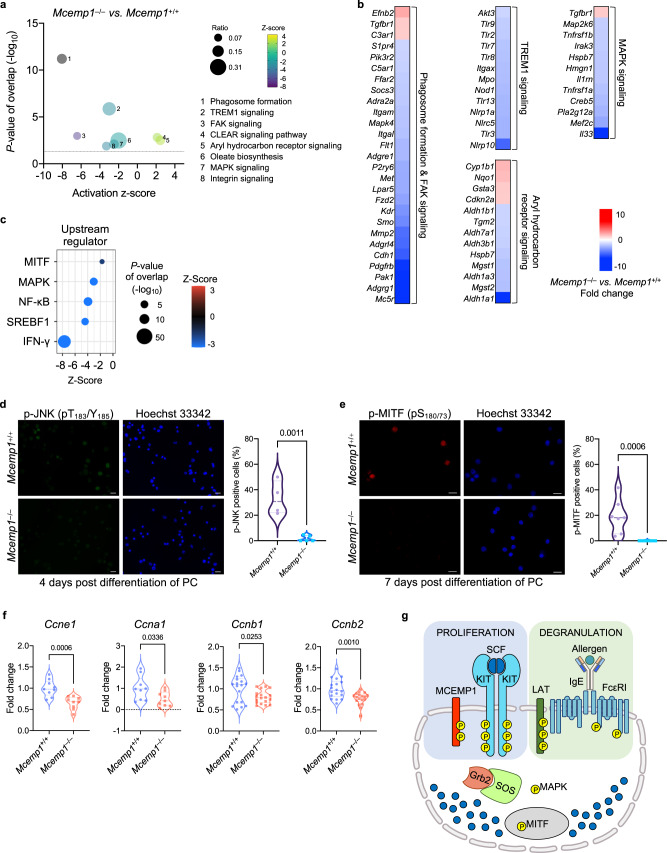
The effect of MCEMP1 deficiency on gene expression related to cell growth. **a** IPA canonical pathways enriched in *Mcemp1*^–/–^ PC versus *Mcemp1*^+/+^ PC cultured with SCF and IL-3 for 9 days. The z-scores indicate a predicted inhibition or activation of the indicated pathway. The ratio was calculated by the number of DEGs in the indicated pathway divided by the total number of genes that map to the pathway. The *p*-value of overlap was calculated by the right-tailed Fisher’s Exact Test. **b** Heatmaps of differentially expressed genes (DEGs) of *Mcemp1*^–/–^ PC versus *Mcemp1*^+/+^ PC. **c** A dot plot of comparison analysis of upstream regulators repressed in *Mcemp1*^–/–^ PC in comparison with *Mcemp1*^+/+^ PC. The color bar indicates z-score of a predicted inhibition in the indicated upstream regulators. The size of each dot corresponds to the p-value of overlap (right-tailed Fisher’s Exact Test). **d**, **e** Representative immunofluorescence images of *Mcemp1*^+/+^ or *Mcemp1*^–/–^ PC cultured with SCF and IL-3 for the indicated days and stained with anti-phospho-JNK antibody (green), anti-phospho-MITF antibody (red) or Hoechst 33342 (blue). Scale bar, 20 μm. The average percentage of p-JNK or p-MITF positive cells per slide (*n* = 4–9 slides) were quantified. Data are representative of two independent experiments. **f** Expressions of *Ccne1*, *Ccna1*, *Ccnb1*, *and Ccnb2* in *Mcemp1*^+/+^ or *Mcemp1*^–/–^ PC. KIT-positive PC were isolated and cultured with SCF and IL-3 for 6 days and stimulated with 30 ng/ml SCF and further cultured for 12 h (*n* = 9-18 per group). **g** Schematic illustration of SCF-KIT and IgE-FcεRI signal transduction in mast cells. The linker for activation of T cells (LAT) is an essential adaptor for FcεRI receptor that induces IgE-mediated mast cell activation. In parallel, MCEMP1 acts as a critical adaptor for KIT receptor that promotes SCF-mediated mast cell proliferation. Data are presented as violin plot with lines at median and quartiles and *p*-values were determined by two-tailed unpaired *t*-test in **d**–**f**. ns, not significant.

Since MCEMP1 expression is involved in mast cell proliferation, we examined the transcriptomic changes in *Mcemp1*^–/–^ PC versus *Mcemp1*^+/+^ PC cultured with SCF and IL-3 for 9 days. *Mcemp1*^–/–^ PC exhibited a total of 1193 downregulated and 340 upregulated genes in comparison to *Mcemp1*^+/+^ PC (Supplementary Fig. 4a). Ingenuity Pathway Analysis (IPA) showed that compared to *Mcemp1*^+/+^ PC, *Mcemp1*^–/–^ PC exhibited downregulation of gene sets associated with phagosome formation/FAK signaling, TREM1 signaling, and MAPK signaling, while demonstrating upregulation of gene sets related to aryl hydrocarbon receptor signaling (Figs. 4a, b). Network IPA identified key differentially expressed genes (DEGs) involved in the reduced proliferation of *Mcemp1*^–/–^ PC (Supplementary Fig. 4b). In addition, IPA upstream analysis identified that signaling molecules (IFN-γ, NF-κB, and MAPK) and transcription factors [SREBF1 and microphthalmia-associated transcription factor (MITF)] were significantly repressed in *Mcemp1*^–/–^ PC compared to *Mcemp1*^+/+^ PC (Fig. 4c). Overall, transcriptomic profiling indicates that deficiency of MCEMP1 significantly disrupts multiple intracellular signaling pathways, leading to a decrease in PC proliferation in vitro.

SCF-mediated KIT receptor activation has been shown to induce downstream signal transduction of MAPK and MITF that facilitates cell proliferation^45–48^. We examined whether MCEMP1 deficiency impairs SCF-mediated MAPK signal transduction and MITF activation in PCMC from *Mcemp1*^+/+^ mice or *Mcemp1*^–/–^ mice. KIT-positive PC were cultured with SCF for 4 to 7 days in vitro. The levels of phosphorylated JNK (T_183_/Y_185_ residues) and MITF (Ser_180/73_ residues) were markedly lower in *Mcemp1*^–/–^ PC when compared to *Mcemp1*^+/+^ PC (Figs. 4d, e). These results indicate that MCEMP1 is required for the efficient phosphorylation and activation of JNK and MITF in response to SCF-mediated KIT receptor activation. In addition, the expression of G1/S-phase cyclins (*Ccne1*, *Ccna1*) and mitotic cyclins (*Ccnb1 and Ccnb2*) was significantly decreased in *Mcemp1*^–/–^ PC compared to *Mcemp1*^+/+^ PC, suggesting that MCEMP1 deficiency results in a reduction of cell cycle progression of peritoneal cell-derived mast cells (Fig. 4f). Collectively, these results suggest that MCEMP1 interacts with and activates KIT activity to induce downstream signal transduction in an ITAM-dependent manner, ultimately facilitating mast cell proliferation (Fig. 4g).

### MCEMP1 deficiency diminishes airway inflammation in chronic asthma mouse model

**Fig. 5:**
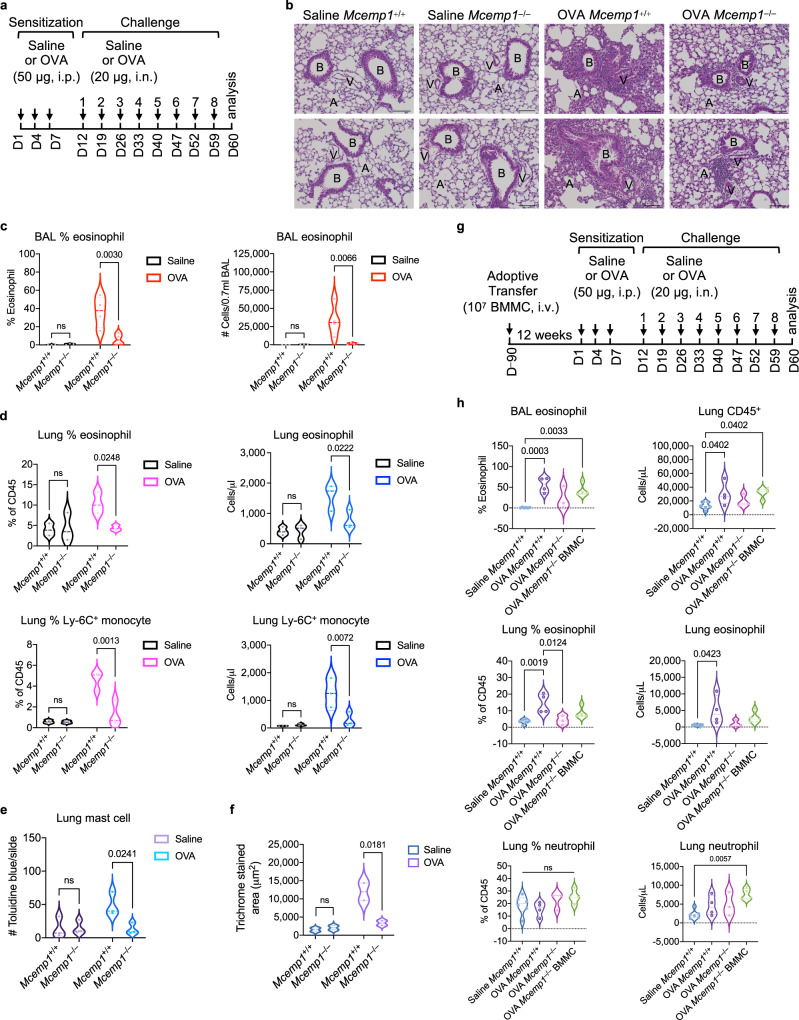
MCEMP1 deficiency attenuates OVA-induced lung inflammation. **a** Schematics of ovalbumin (OVA)-sensitized and challenged chronic asthma model. *Mcemp1*^+/+^ and *Mcemp1*^–/–^ mice were sensitized with saline or 50 μg OVA via intraperitoneal (i.p.) injection and challenged with saline or 20 μg OVA via intranasal (i.n.) instillation on the indicated days. **b** Representative images of hematoxylin and eosin staining for histological analysis of lungs. B, bronchiole; A, alveoli; V, blood capillary. Scale bar, 100 μm. **c** Percentages and absolute numbers of eosinophils in bronchoalveolar lavage (BAL) were determined by manual counting and by differential counts on cytospin slides (*n* = 3 for saline group, *n* = 4 for OVA group). **d** Percentages and absolute numbers of eosinophils and Ly-6C^+^ inflammatory monocytes in the lungs were determined by flow cytometry (*n* = 3 per group). **e** Average numbers of toluidine blue positive mast cells per slide (*n* = 3 slides per group) were quantified by ImageJ. **f** Quantification of Masson’s trichrome staining (*n* = 2 slides per group) by Image-Pro 10. **g** Schematics of BMMC adoptive transfer and OVA-induced chronic asthma model. **h** Percentages and/or absolute numbers of BAL eosinophils, lung CD45^+^ leukocytes, eosinophils and neutrophils were determined by flow cytometry (*n* = 6 for saline group. *n* = 3-4 for OVA group). Data are presented as violin plots with lines at median and quartiles and *p*-values were determined by two-way ANOVA with Sidak’s multiple comparison in **c**, **d**, **e**, **f** or by one-way ANOVA with Tukey’s multiple comparison in **h**. ns, not significant.

Several studies have shown that MCEMP1 is one of the top inducible genes in inflammatory lung diseases including asthma^2–4^. Indeed, when C57BL/6 mice were sensitized with chicken egg ovalbumin (OVA) via intraperitoneal injection and subsequently challenged with the same antigen via intranasal administration, MCEMP1 expression was remarkably upregulated in the lung lesions, but not in the guts (Supplementary Fig. 4c, d). To examine whether MCEMP1 deficiency impairs the immune response in chronic asthma model, 6–8 weeks old *Mcemp1*^+/+^ or *Mcemp1*^–/–^ C57BL/6 mice were immunized via intraperitoneal injection of OVA on days 1, 4, and 7 (Fig. 5a). At 5 days after the third sensitization, mice were challenged with intranasal instillation of OVA weekly for a total of 8 weeks. Control mice received saline for both sensitization and challenge. Compared to saline-treated *Mcemp1*^+/+^ and *Mcemp1*^–/–^ mice, OVA-challenged *Mcemp1*^+/+^ mice exhibited significant peribronchiolar and perivascular leukocyte infiltration and lung damage, which was considerably reduced in OVA-challenged *Mcemp1*^–/–^ mice (Fig. 5b). Strikingly, *Mcemp1*^–/–^ mice showed lower percentages and numbers of eosinophils in bronchoalveolar lavage (BAL) fluid than *Mcemp1*^+/+^ mice after being challenged with OVA (Fig. 5c). Moreover, the percentages and absolute numbers of infiltrated eosinophils and inflammatory monocytes, but not neutrophils, were considerably lower in the lungs of *Mcemp1*^–/–^ mice than *Mcemp1*^+/+^ mice upon OVA challenge (Fig. 5d and Supplementary Fig. 5a, b). In addition, *Mcemp1*^–/–^ mice showed decreased levels of mast cells accumulation in the lungs compared to *Mcemp1*^+/+^ mice after being challenged with OVA (Fig. 5e and Supplementary Fig. 5c). In comparison to OVA-challenged *Mcemp1*^+/+^ mice, OVA-challenged *Mcemp1*^–/–^ mice also exhibited less collagen deposition in the lungs, indicating reduced lung fibrosis (Fig. 5f and Supplementary Fig. 5d). These findings indicate that MCEMP1 deficiency leads to the mitigation of OVA-induced chronic inflammatory responses, such as eosinophil and inflammatory monocyte infiltration, mast cell accumulation, as well as collagen deposition.

Furthermore, we performed mast cell engraftment into *Mcemp1*^–/–^ mice for OVA-induced chronic lung inflammation. Bone marrow cells from *Mcemp1*^+/+^ mice were cultured with SCF and IL-3 for 4 weeks. 10^7^ bone marrow-derived WT mast cells were intravenously injected into 10-week-old recipient mouse via the tail vein. At 12 weeks later, *Mcemp1*^–/–^ recipients were subjected to OVA-induced chronic asthma model (Fig. 5g). Consistently, the percentages and absolute numbers of infiltrated eosinophils and inflammatory monocytes were considerably higher in the lungs of *Mcemp1*^+/+^ mice than *Mcemp1*^–/–^ mice after being challenged with OVA (Fig. 5g and Supplementary Fig. 5e). However, adoptive transfer of WT mast cells into *Mcemp1*^–/–^ mice led to the increase of OVA-induced infiltrates of leukocytes including eosinophils and neutrophils, and the decrease of percentages of alveolar macrophages compared to saline-treated *Mcemp1*^+/+^ mice or OVA-treated *Mcemp1*^–/–^ mice (Fig. 5h and Supplementary Fig. 5e). These results indicate that MCEMP1 expression in mast cells is critical for the OVA-induced recruitment of leukocytes including eosinophils and neutrophils in chronic asthma model.

### MCEMP1 deficiency alters gene expression related to lung inflammation and damage

**Fig. 6:**
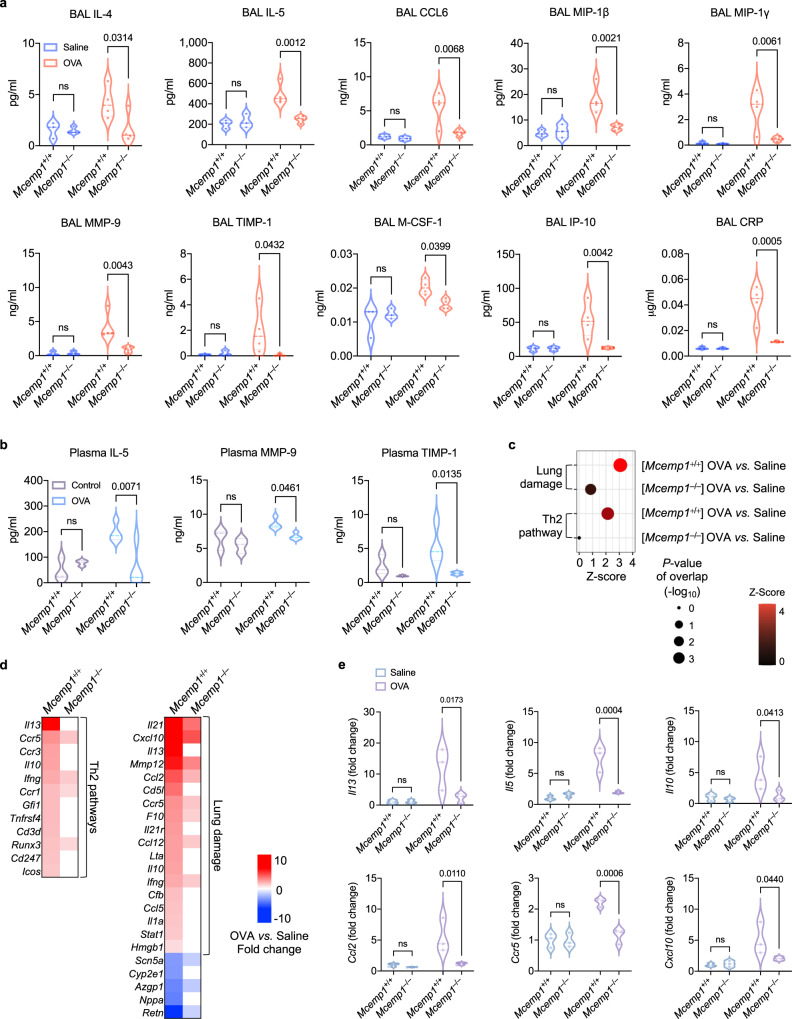
MCEMP1 deficiency diminishes asthma-associated inflammatory gene expression. **a**, **b** Cytokine and growth factor levels in the BAL and plasma were determined by multiplex analysis (*n* = 3 for saline group, *n* = 4 for OVA group). **c** A dot plot of comparison analysis of OVA-challenged versus saline-treated *Mcemp1*^+/+^ mice in comparison with OVA-challenged versus saline-treated *Mcemp1*^–/–^ mice. The color bar indicates z-score of a predicted activation in the indicated pathway. The size of each dot corresponds to the *p*-value of overlap (right-tailed Fisher’s Exact Test). **d** Heatmaps of significantly upregulated and downregulated genes in OVA-challenged versus saline-treated *Mcemp1*^+/+^ mice in comparison with OVA-challenged versus saline-treated *Mcemp1*^–/–^ mice. Red, white, and blue indicate induction, no change, and repression, respectively. **e** Gene expression of *Il13*, *Il5*, *Il10*, *Ccl2*, *Ccr5*, and *Cxcl10* in *Mcemp1*^+/+^ mice or *Mcemp1*^–/–^ mice after saline or OVA challenge. Data are presented as violin plots with lines at median and quartiles and *p*-values were determined by two-way ANOVA with Sidak’s multiple comparison in **a**, **b**, **e**. ns, not significant.

Furthermore, we assessed multiple cytokines and growth factors in the BAL and plasma. *Mcemp1*^–/–^ mice exhibited significantly lower levels of BAL concentrations of Th2-type cytokines (IL-4 and IL-5), chemokines and growth factors [CCL6, MIP-1γ (CCL9), KC-GRO (CXCL1), M-CSF-1, IP-10 (CXCL10), MIP-1β (CCL4)], airway remodeling-related molecules (MMP-9 and TIMP-1), and inflammatory substances (CRP, RAGE, VEGF-A, and VCAM-1) for airway obstruction and chronic vascular inflammation, compared to *Mcemp1*^+/+^ mice upon OVA challenge (Fig. 6a and Supplementary Fig. 6a). In addition, OVA-challenged *Mcemp1*^–/–^ mice showed lower concentrations of plasma IL-5, MMP-9, and TIMP-1 than OVA-challenged *Mcemp1*^+/+^ mice (Fig. 6b). However, *Mcemp1*^+/+^ and *Mcemp1*^–/–^ mice showed similar amounts (100 to 150 pg/mL) of SCF in bronchoalveolar lavages, suggesting that the low proliferation of mast cells in *Mcemp1*^–/–^ mice is not due to the low level of SCF but due to the lack of MCEMP1-mediated activation of KIT (Supplementary Fig. 6a). Collectively, these data indicate that MCEMP1 deficiency results in the reduction of proinflammatory cytokine-mediated airway inflammation.

We further analyzed the lung transcriptome of OVA-challenged mice by RNA sequencing. The number of DEGs in OVA-challenged *Mcemp1*^+/+^ mice compared to saline-treated *Mcemp1*^+/+^ mice was significantly higher than those in OVA-challenged *Mcemp1*^–/–^ mice compared to saline-treated *Mcemp1*^–/–^ mice (Supplementary Fig. 6b). The extent of gene expression alteration between OVA-challenged versus saline-treated *Mcemp1*^+/+^ mice was much greater than that between OVA-challenged versus saline-treated *Mcemp1*^–/–^ mice (Supplementary Fig. 6c). In addition, OVA challenge led to greater upregulation of genes related to Th2 pathway and lung damage in *Mcemp1*^+/+^ mice compared to *Mcemp1*^–/–^ mice (Fig. 6c). Among the upregulated core DEGs in OVA-challenged versus saline-treated *Mcemp1*^+/+^ mice were genes encoding Th2-type cytokines and cytokine receptors (*Il13*, *Il10*, *Ccl5*, *Ccr1*, *Ccl3*, and *Ccr5*), interferon related genes (*Ifng* and *Stat1*), matrix metallopeptidase (*Mmp12*), and complement proteins (*C3* and *Cfb*) (Fig. 6d). In contrast, the alteration of these gene expressions was markedly reduced in *Mcemp1*^–/–^ mice (Fig. 6d). qRT-PCR analysis of lung tissues further showed that OVA-challenged *Mcemp1*^–/–^ mice had lower expression levels of *Il13*, *Il5*, *Il10*, *Ccl2*, *Ccr5*, and *Cxcl10* compared to OVA-challenged *Mcemp1*^+/+^ mice (Fig. 6e). Taken together, these data indicate that MCEMP1 deficiency results in a global suppression of various lung-specific signaling pathways, with a particular impact on those related to airway inflammation and lung damage.

### MCEMP1 deficiency alleviates lung function impairment in OVA-induced chronic asthma

**Fig. 7:**
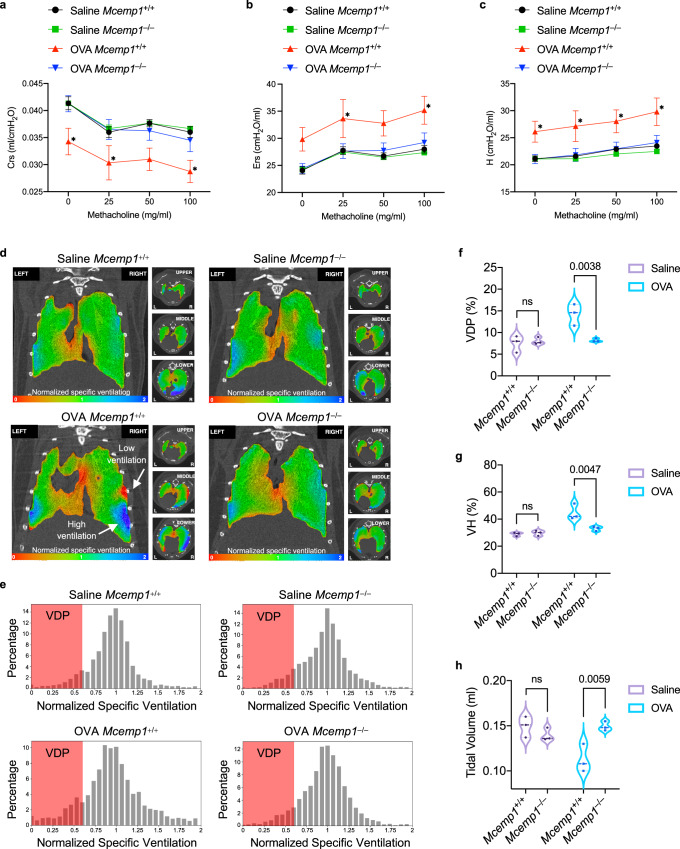
MCEMP1 deficiency ameliorates lung compliance and airflow in OVA-induced chronic asthma. **a**–**c** Lung function parameters; compliance (Crs), elastance (Ers) of respiratory system, and tissue elastance (H) in response to the indicated concentrations of methacholine were measured 1 day after the eighth intranasal challenge with OVA or saline (*n* = 3-4 per group). **d** Representative images of 4DMedical lung scanning for regional ventilation visualization across the lung over a full inhalation. One coronal slice and three axial slices of upper, middle, and lower lungs of *Mcemp1*^+/+^ or *Mcemp1*^–/–^ mice challenged with OVA or saline. The color bar indicates the degree of ventilation as low in red and high in blue. **e** Representative histogram of 4DMedical ventilation frequency distribution. VDP, ventilation deficit percent. **f**–**h** Ventilation metrics; VDP, ventilation heterogeneity (VH), and tidal volume (*n* = 3 per group). Data are presented as mean±s.d. in **a**, **b**, **c** and as violin plots with lines at median and quartiles in **f**, **g**, **h**. *P*-values were determined by two-way ANOVA with Dunnett’s multiple comparison in **a**, **b**, **c** and by two-way ANOVA with Sidak’s multiple comparison in **f**, **g**, **h**. ^*^P < 0.05 (Crs, the *p*-values for 0, 25, and 100 mg/ml methacholine are 0.0115, 0.034, and 0.0444, respectively; Ers, the *p*-values for 25 and 100 mg/ml methacholine are 0.0442 and 0.0286, respectively; H, the *p*-values for 0, 25, 50, and 100 mg/ml methacholine are 0.0361, 0.0425, 0.0346, and 0.0157, respectively). ns, not significant.

We next examined lung function parameters of OVA-challenged mice, including lung stiffness and airway hyperreactivity, upon methacholine inhalation using Flexivent system. OVA-challenged *Mcemp1*^+/+^ mice exhibited significantly decreased compliance of respiratory system (Crs, ability of the lung to stretch) in response to increasing doses of methacholine, whereas OVA-challenged *Mcemp1*^–/–^ mice showed similar levels of Crs to saline-treated *Mcemp1*^+/+^ or *Mcemp1*^–/–^ mice (Fig. 7a). OVA-challenged *Mcemp1*^+/+^ mice also showed increased elastance of respiratory system (Ers, an index of stiffness) and tissue elastance (H, alveoli stiffness) compared to OVA-challenged *Mcemp1*^–/–^ mice, indicating that *Mcemp1*^–/–^ mouse lung was less remodeled and stiffer than *Mcemp1*^+/+^ mouse lung when intranasally challenged with OVA (Figs. 7b, c). On the other hand, OVA-challenge did not significantly increase the overall resistance of the respiratory system (Rrs), and central (Rn) and alveoli airway (G) in either *Mcemp1*^+/+^ mice or *Mcemp1*^–/–^ mice (Supplementary Fig. 7a, b, c).

We further assessed functional lung impairments using X-ray velocimetric 4-dimensional computed tomography (4DMedical lung scanning) technology that allows the visualization of lung motion and quantification of time-resolved regional airflow. OVA-challenged *Mcemp1*^+/+^ mice exhibited sporadic distributions of increased or decreased regional ventilation the peak of inspiration as represented by the blue or the red area, respectively, whereas OVA-challenged *Mcemp1*^–/–^ mice displayed a relatively uniform regional ventilation (Fig. 7d). The ventilation frequency distribution histogram showed that OVA-challenged *Mcemp1*^+/+^ mice had a significantly higher ventilation deficit percent (VDP), defined as the percentage of ventilation less than 60% of the mean, compared to OVA-challenged *Mcemp1*^–/–^ mice, saline-treated *Mcemp1*^+/+^ mice, or saline-treated *Mcemp1*^–/–^ mice (Figs. 7e, f). Consistently, the ventilation heterogeneity (VH) of OVA-challenged *Mcemp1*^+/+^ mice was higher than OVA-challenged *Mcemp1*^–/–^ mice regardless of the small or large region of airways (Fig. 7g and Supplementary Fig. 7d, e). Tidal volume, which is the total volume of air inhaled and exhaled in each breath, was significantly lower in OVA-challenged *Mcemp1*^+/+^ mice compared to OVA-challenged *Mcemp1*^–/–^ mice (Fig. 7h). These results indicate that *Mcemp1*^–/–^ mice have significantly weaker functional ventilation defect phenotypes than *Mcemp1*^+/+^ mice following OVA challenge. Overall, Flexivent lung functional examinations and 4DMedical imaging results indicate that MCEMP1 deficiency leads to reduced lung inflammation and improved lung function in OVA-induced chronic asthma model.

## Discussion

Our study implicates the critical function of MCEMP1 as an adaptor for the KIT receptor in promoting SCF-mediated mast cell proliferation, which in turn contributes to chronic airway inflammation. The crystal structure of KIT has alluded that the intracellular domain of KIT features a high-order regulation of its kinase activity through multiple post-translational modifications and conformational changes^31,32^. For instance, the JM domain of KIT excerts an autoinhibitory function by interacting with the kinase domains and suppressing its kinase activity^32^. Upon SCF-mediated KIT activation, two tyrosine residues in the JM domain of KIT undergo phosphorylation that releases its autoinhibitory configuration, allowing the two kinase domains to adopt a conformation for active catalytic function^32^. We found that the deletion of JM and KD1 regions of KIT led to a loss of binding to MCEMP1, suggesting that MCEMP1 may interact with either the JM or KD1 domain of KIT and interfere with its autoinhibitory configuration and keeps the kinase domain of KIT in an active form. Human MCEMP1 shares 44% amino acid sequence identity with mouse MCEMP1 ortholog. A cross-species interaction study demonstrated that human MCEMP1 interacts with mouse KIT receptor, suggesting the presence of an evolutionarily conserved motif or similar secondary structure within the intracellular domain of MCEMP1 that is essential for KIT binding. Further investigation is required to validate this hypothesis and elucidate the molecular mechanism of MCEMP1-KIT interaction in KIT activation.

Moreover, our study demonstrates that MCEMP1 is critical for optimal proliferation of primary mast cell populations in the peritoneum, but not in bone marrow. Our in vitro study indicates that MCEMP1 is critical for SCF/KIT-mediated mast cell proliferation, indicating that MCEMP1 may primarily be responsible for SCF-dependent signaling, rather than IL-3-dependent signaling, for mast cell growth. This is further supported by our in vivo study demonstrating impaired lung mast cell expansion in *Mcemp1*-deficient mice upon intranasal SCF challenge. Overall, our study elucidates a regulatory mechanism that accounts for KIT-mediated mast cell proliferation.

We have also evaluated the pathological immune responses in the airways of *Mcemp1*^–/–^ mice after sensitization and challenge to OVA. We developed a chronic asthma mouse model in *Mcemp1*^–/–^ mice of the C57BL/6 strain, based on previous studies that utilized C57BL/6-Kit^W-sh^ mast cell-deficient mice to demonstrate the critical role of mast cells in the OVA-induced chronic asthma model^36,49^. MCEMP1 deficiency resulted in significantly lower levels of inflammatory cytokines, decreased numbers of eosinophils and mast cells, and less deposition of fibrotic collagen in the lung. We employed Flexivent to measure airway responsiveness in response to methacholine and 4DMedical lung scanning to assess respiratory mechanics. Combination of these advanced technologies enabled us to analyze various lung parameters and draw phenotypical consequences of MCEMP1 deficiency. Compared to *Mcemp1*^+/+^ mice, *Mcemp1*^–/–^ mice developed less affected lung functions, including lung compliance, elastance, ventilation heterogeneity, ventilation deficit percent, and tidal volume. Mechanistically, our in vivo study indicates that MCEMP1 promotes the expansion of mast cells, which contributes to severe lung inflammation in asthma. RNA-seq analysis of lung tissues showed that *Mcemp1*^–/–^ mice exhibited downregulation of gene expressions related to lung damage in comparison to *Mcemp1*^+/+^ mice, supporting the notion that MCEMP1 is a crucial factor that mediates immune responses and airway inflammation in the development of chronic asthma.

Mast cells originate from hematopoietic progenitor cells in the bone marrow. Immature progenitor cells that circulate in the blood migrate to the periphery, where they further differentiate into mature mast cells^50–52^. Tissue-resident mast cells exhibit phenotypic heterogeneity (maturity, granule formation, and varied mast cell mediators) and considerable plasticity that are influenced by microenvironmental factors such as cytokines, growth factors, and microbiome^13,27,28,53,54^. Further investigation of lung mast cell phenotypes from *Mcemp1*^–/–^ mice in chronic asthma model is required to examine whether *Mcemp1*^–/–^ mice have defects in the development of distinct mast cell subtype clusters, induction of specific gene sets or pathways that contribute to asthma exacerbation in comparison with *Mcemp1*^+/+^ mice upon OVA challenge. It is important to include different mouse strains in further studies as the genetic background can have an impact on the functional properties of mast cells. MCEMP1 and KIT are also expressed in other immune cells including dendritic cells and hematopoietic progenitor cells and KIT signaling is also involved in mast cell proliferation, differentiation, survival, and migration. Thus, further studies are required to gain a better understanding of the role and mechanism of action of MCEMP1 in other aspects of mast cell biology, tissue-specific mast cells, additional immune cells, its potential ligand(s), and protein kinases responsible for MCEMP1 phosphorylation.

Therapeutic interventions aimed at modulating mast cell functions can be achieved by various approaches, including reducing the mast cell population, inhibiting mast cell activation, intervening intracellular signal transduction, and blocking the effects of mast cell mediators^12^. A comprehensive understanding of the signaling pathways and mechanisms that contribute to mast cell hyper-proliferation or hyperplasia in human tissue is necessary for the development of targeted therapies aimed at regulating mast cell numbers. Reducing mast cell numbers is a promising treatment strategy for clonal mast cell disorders such as mastocytosis and allergic lung diseases, which are characterized by abnormally elevated mast cell populations. The SCF-mediated KIT receptor signaling is considered as a critical factor for mast cell hyperplasia in physiological and pathological conditions. Given that MCEMP1 expression is highly upregulated in allergic and inflammatory lung diseases^2–4^ and that MCEMP1 regulates SCF/KIT-mediated mast cell proliferation, the development of MCEMP1 inhibitors holds significant potential for the treatment of lung inflammatory diseases. In summary, this study advances our understanding of the MCEMP1-mediated regulation of KIT receptor for mast cell proliferation and asthma development, and further offer molecular insights into the development of new therapies for allergic and inflammatory lung diseases.

## Methods

All experiments were reviewed and approved by Institutional Biosafety Committee (IBC) and Institutional Animal Care and Use committees (IACUC) at USC and Cleveland Clinic.

### Cell culture

293T, Raw264.7, HMC-1 cells were purchased from ATCC. C57 and DC2.4 cells were kindly provided by S.J. Galli and K.L. Rock, respectively. 293T and RAW264.7 cell lines were grown in DMEM (Gibco, 11965-092) supplemented with 10% fetal bovine serum (FBS, Gibco, 26140-079), 100 U/ml penicillin, and 100 μg/ml streptomycin (1% Pen/Strep, Gibco, 15140-163). C57 cells were cultured in DMEM supplemented with 10% FBS, 1% Pen/Strep, 2 mM L-glutamine (Gibco, 35050-061), and 55 μM β-mercaptoethanol (β-ME, Gibco, 21985-023). HMC-1 cells were culture in RPMI-1640 (Gibco, 11875-093) supplemented with 10% FBS, 1% Pen/Strep, and 55 μM β-ME. DC2.4 cells were cultured in RPMI-1640 supplemented with 10% FBS, 1% Pen/Strep, 2 mM L-glutamine, 1× non-essential amino acids (Gibco, 11140-050), 10 mM HEPES pH 7.5 (Gibco, 15630-080), and 55 μM β-ME. All cells were maintained at 37 °C with 5% CO_2_.

### Plasmids

All constructs for transient and stable expression in mammalian cells include pEF-MCS-IRES-puro (modified), pCDH-CMV-MCS-EF1-puro (System Biosciences, CD510B-1), pCDH-CMV-MCS-EF1-Hygro (System Biosciences, CD515B-1), and pLKO.1-TRC (Addgene, 10878) vectors. All human and mouse MCEMP1 expression plasmids contain a carboxy-terminal Flag epitope and all human and mouse KIT expression plasmids contain a carboxy-terminal HA or V5 epitope. All constructs were constructed by standard PCR cloning strategy or Gibson assembly method.

### Co-immunoprecipitation and GST-pulldown

293T cells were transfected with the indicated DNA plasmids using polyethylenimine transfection (Polysciences, 23966). Cells were collected 48 h post-transfection, washed by DPBS pH 7.5 (Gibco, 14190-144), and resuspended in 1% Triton X-100 lysis buffer containing 50 mM Tris-HCl pH 8.0 (Invitrogen, 15568-025), 150 mM NaCl (Sigma, 31434), 1% Triton X-100 (Sigma, T9284), supplemented with protease inhibitor cocktail (Roche, 48047900). After sonication or freeze/thaw cycles, whole-cell lysates (WCL) were rotating at 4 °C for 1 h and centrifuged for 10 min at 16,000 × *g*. The supernatants were filtered through a 0.45 μm polyethersulfone filter. For Co-immunoprecipitation, WCL were incubated with M2 beads (Sigma, F2426) or HA beads (Sigma, E6779) at 4 °C for 1-2 h. For GST-pulldown, WCL were incubated with glutathione-conjugated sepharose beads (GE, GE17-0756-01) at 4 °C for 1 h. The immobilized M2 immune complexes or GST complexes containing beads were washed five times using 1% Triton X-100 lysis buffer containing 150~400 mM NaCl. Beads were eluted in 2×Laemmli dye (Sigma, S3401) by heating at 95 °C for 5 min and subjected to immunoblotting.

### Immunoblotting

Antibodies included MCEMP1 (polyclonal rabbit made by GST-mouse MCEMP1-ectodomain fusion protein), Phospho-KIT (Cell Signaling, 3391S), KIT (Cell Signaling, 3074S), SOS1 (Cell Signaling, 12409S), Grb2 (Santa Cruz, C-23), 4G10 (EMD Millipore, 05-321), 4G10-HRP (EMD Millipore, 16-105), P-Tyr-1000 (Cell Signaling, 8952S), Phospho-IκBα (Cell Signaling, 9241S), IκBα (Cell Signaling, 4814S), Phospho-MEK1/2 (Cell Signaling, 9154S), MEK1/2 (Cell Signaling, 9122S), Phospho-p38 (Cell Signaling, 9211S), p38 (Cell Signaling, 9212S), Phospho-JNK (Cell Signaling, 9251S), JNK (Cell Signaling, 4668S), Phospho-ERK (Cell Signaling, 4370S), ERK (Cell Signaling, 4695S or 9101S), GST (Santa Cruz, B-14), Actin (Santa Cruz, C4), Tubulin (Santa Cruz, B7), rabbit-Flag (Sigma, F7425), mouse-Flag (Sigma, F1804, M2), rabbit-HA (Covance, PRB-101P and BioLegend 902302), mouse-HA (BioLegend 901503, 16B12), mouse Flag-HRP (Abcam ab49763, M2), mouse HA-HRP (BioLegend 901519, 16B12), mouse V5-HRP (Thermo R961-25), mouse IgG-HRP (Cell Signaling, 7076), and rabbit IgG-HRP (Cell Signaling, 7074). Information on antibodies for immunoblotting is presented in Supplementary Table 2.

### Flag antibody stimulation

Cells (5 × 10^6^) were washed once with serum free DMEM, resuspended in 25 μl serum free DMEM, pre-incubated for 10 min at 37 °C. 25 μl of cells were incubated with 25 μl of monoclonal mouse anti-Flag antibody (1 μg) for 1~5 min at 37 °C. Cells were centrifuged at 1000 × *g* for 1 min at 4 °C and rapidly frozen by a dry ice/ethanol or liquid nitrogen. Cells were lysed with 1 ml of 1% Triton X-100 lysis buffer containing 50 mM Tris-HCl pH 8.0, 150 mM NaCl, 1% Triton X-100, supplemented with protease inhibitor cocktail, phosphatase inhibitor cocktail (Roche 04906 837001), and 2 mM Na_3_VO_4_ (tyrosine phosphatase inhibitor, NEB P0758L). WCL were immunoblotted with indicated antibodies. To detect the phosphorylated MCEMP1, WCL were incubated with rabbit anti-Flag antibody at 4 °C for 3–12 h, followed by incubation with protein A/G agarose beads (Thermo Fisher, 20422) at 4 °C for 3 h. The immobilized immune complexes were washed five times with 1% Triton X-100 lysis buffer containing 150 to 300 mM NaCl. Beads were eluted in 2×Laemmli dye by heating at 95 °C for 5 min and subjected to immunoblotting with 4G10 antibody. To perform a qRT-PCR, cells (10^6^) were incubated with 1 μg/ml anti-Flag antibody for 6–8 h at 37 °C.

### qRT-PCR

500 ng to 1 μg total RNA was extracted using TRI reagent (Sigma, T9424) or RNeasy kit (Qiagen, 74104 or 74004), digested with DNase I (Sigma, AMPD1), and reverse-transcribed into cDNA using iScript cDNA synthesis kit (Bio-Rad, 1708891). cDNAs were quantified using iQ SYBR Green Supermix kit (Bio-Rad, 1725271) and a CFX96 thermocycler (Bio-Rad). Gene-specific probes for qRT-PCR are listed in Supplementary Table 3.

### Calcium mobilization assay

Cells (2 × 10^6^) were loaded with calcium indicator (BD, 640176) in 200 μl of complete RPMI-1640 medium for 30 min at 37 °C, washed once, resuspended in 1 ml of the medium, and put on ice until analyzed. Baseline calcium level was established for 1 min prior to addition of stimuli. Cells were stimulated with 1 μg/ml anti-Flag antibody or 2 μM calcium Ionophore A23187 (Sigma, C7522), then intracellular calcium level was monitored by flow cytometry analysis.

### Degranulation assay

Cells (10^6^) were preloaded with 100 ng/ml anti-2,4,dinitrophenyl antibody (α-DNP IgE, Clone SPE-7, Sigma, D8406) at 37 °C for 18 h. Cells were washed twice and resuspended in 250 μl of Tyrode’s buffer pH 7.4 containing 10 mM HEPES, 129 mM NaCl, 5 mM KCl, 1.4 mM CaCl_2_, 1 mM MgCl_2_, 5.6 mM Glucose, 0.1% BSA. 10 μl of cells were incubated with 10 μl of 2,4,dinitrophenyl -BSA (DNP-BSA, 2-200 ng/ml, Invitrogen, A23018), anti-Flag antibody (1 μg/ml, Sigma, F7425), anti-V5 antibody (1 μg/ml, Invitrogen, 46-1157), phorbol 12-myristate 13-acetate (PMA, 50 ng/ml, Sigma, P8139) plus calcium ionophore A23187 (10 μM) in 96-well V-bottom plate at 37 °C for 1 h without CO_2_. Cells were centrifuged at 500 × *g* for 3 min and supernatant (10 μl) was transferred to a new 96-well flat-bottom plate. Cell pellets were lysed with 40 μl of 1% Triton X-100 in Tyrode’s buffer. After centrifugation at 500 × *g* for 3 min to remove debris, cell lysate (10 μl) was transferred to the 96-well flat-bottom plate. The substrate 4-nitrophenyl N-acetyl-β-D-glucosaminide (p-NAG, 4 mM, Sigma, N9376) was prepared in a substrate buffer pH 4.5 containing 50.3 mM Na_2_HPO_4_ (Sigma, S7907) and 109.4 mM citric acid (Sigma, 251275). The 10 μl of supernatant and cell lysate were incubated with 40 μl of *p*-NAG at 37 °C for 90 min without CO_2_. Reactions were stopped with 150 μl of 0.2 M glycine pH 10.7 (Bio-Rad, 1610724) and read at 405 nm using Filtermax F5 plate reader (Molecular Devices).

### *TKX1* expression system and mass spectrometry

GST fusion proteins were purified from *E. coli* strain *TKX1*, which contains a mammalian elk tyrosine kinase expression vector (Agilent, 200124) as previously described^55^. Briefly, the *TKX*1 strain was transformed with a pGEX6p-1 plasmids expressing 5’GST-tagged human wild-type MCEMP1 or mutant MCEMP1 amino-terminal intracellular domain. The phosphorylated MCEMP1 proteins were purified by GST-pulldown. HMC-1 human mast cell lysates were incubated with the glutathione beads containing GST fusion proteins in a binding buffer containing 20 mM HEPES pH 7.4, 100 mM NaCl, 1% NP-40 (Sigma, 74385), and protease inhibitors at 4 °C for 2 h. Glutathione beads were then washed four times with binding buffer, and the proteins associated with the beads were analyzed by SDS-PAGE and subjected to silver staining. Protein bands were excised and sent to Taplin Biological Mass Spectrometry Facility at Harvard Medical School (HMS) for protein identification.

### SCF purification for in vivo study

pCMV3-sp-HA-mKITLG plasmid (Sino Biological) was used to construct pET21b-mouse-SCF vector. The construct was transformed and expressed in *E. coli BL21 (DE3) RIPL* strain (Agilent, 230280). Cells were grown in LB broth (Invitrogen, 12780-052) containing ampicillin (Sigma, A9518) and chloramphenicol (Sigma, C0378) at 37 °C until OD_600_ reached 0.8 and the protein expression was induced with 1 mM Isopropyl β-d−1-thiogalactoside (GoldBio, 367-93-1) at 37 °C for 4 h. Bacteria were lysed in a buffer containing 10 mM Tris-HCl pH 8.0, 150 mM NaCl, 1% Triton X-100, 0.01 mM EDTA pH 8.0 (Invitrogen, 15575-038), and 1 mM DL-Dithiothreitol (DTT; GoldBio, DTT100). After sonication, cell lysates were centrifuged at 16,000 × *g* for 15 min at 4 °C. Inclusion body pellet was washed three times with lysis buffer (wash buffer I), and centrifuged at 16,000 × *g* for 15 min at 4 °C. Inclusion body pellet was washed again with wash buffer II containing 10 mM Tris-HCl pH 8.0, 150 mM NaCl, 0.01 mM EDTA, and 1 mM DTT to remove Triton X-100, and centrifuged at 16,000 × *g* for 15 min at 4 °C. Inclusion body pellet was solubilized in a dissolving buffer containing 8 M urea (Sigma, U5378), 50 mM sodium acetate pH 6.0 (Sigma, S2889), 0.1 mM EDTA, and 1 mM DTT. The solubilized SCF was slowly injected into the vortex of stirring refolding buffer containing 2.5 M urea, 0.01 mM EDTA, 5 mM sodium acetate, 50 mM Tris-HCl pH 8.5, 0.5 mM oxidized glutathione (Sigma, G4501), 5 mM reduced glutathione (Sigma, G4251), and 0.2 mM phenylmethanesulfonylfluoride (Sigma, 78830) by slowly dripping from 21 G needle at 4 °C. After 40 h, the refolding mixture was dialyzed in a dialysis buffer containing 10 mM Tris-HCl pH 8.0 and 150 mM NaCl. The dialyzed solution was subjected to 30% followed by 70% ammonium sulfate (Sigma, A4418) precipitation to remove misfolded aggregates and enrich for properly folded proteins. The 70% precipitates were resolubilized in 1 X HEPES buffered saline (HBS) and purified by HisPur cobalt resin (Thermo Fisher, 89965) and size exclusion chromatography with a Superdex-200 column (GE, 28990945) using HBS buffer. For in vivo experiment, endotoxin was removed using Triton X-114 (Sigma, X114) and endotoxin removal was confirmed using the ToxinSensor Chromogenic LAL Endotoxin Assay Kit (Genscript, L00350C).

### Mice

*Mcemp1*^–/–^ mice (C57BL/6) were generated by CRISPR-Cas9 system. For production of sgRNA, bicistronic vector expressing Cas9 and sgRNA were digested with BsmBI and the linearized vector was gel purified^56^. MCEMP1-targeting guide RNA sequence (*Mcemp1*-RGEN) was kindly provided by J.-S. Kim^57^. A pair of oligos for target exon 1 of mouse *Mcemp1* was annealed, phosphorylated, and ligated into linearized vector. T7 promoter was then added to the sgRNA template by PCR amplification. Gel-purified T7-sgRNA PCR product was used as a template for in vitro transcription using MEGAshortscript T7 Transcription kit (Life Technologies, AM1354), subsequently purified by MEGAclear kit (Life Technologies, AM1908) and eluted in RNase-free water. Microinjection of the oligos was performed by the University of Southern California (USC) transgenic/knockout rodent core facility. Briefly, *Cas9* mRNA (TriLink, L-6129) and sgRNA were mixed and injected into pronuclei-stage zygotes obtained from the embryo donor C57BL/6 female mice. The injected zygotes were transferred into the uterus of pseudopregnant B6D2F1 (C57BL/6 X DBA2) foster female mice. For genotyping of pups, genomic DNA was extracted from the tail and the sgRNA target site was PCR amplified using the following primers (Forward: 5’-TGAGATTTCCCCATCTATCTGA-3’; Reverse: 5’- CTCACCTTGTTTTGTGGGTGT-3’). The PCR products were then subjected to T7 endonuclease I assay (NEB, M0302L) and Sanger sequencing. All mice were bred and housed in specific pathogen-free facilities maintained by the USC Animal Research Center at Keck Medical School and Cleveland Clinic Lerner Research Institute. All animal experiments were reviewed and approved by Institutional Animal Care and Use committees (IACUC) at USC and Cleveland Clinic.

### Primary mast cell and macrophage isolation

Peritoneal cells (PC) were isolated using a buffer containing HBSS (Gibco, 14175-095), 10 mM HEPES pH 7.5, and 3% FBS. Bone marrow cells (BM) were isolated from femur and tibia using RPMI-1640. PC and BM were grown in RPMI-1640 media supplemented with 10% FBS, 1% Pen/Strep, 2mM L-glutamine, 1 mM sodium pyruvate (Thermo Fisher, 11360-070), 10 mM HEPES pH 7.5, 55 μM β-ME, 30 ng/ml SCF (Peprotech, 250-03) and/or 30 ng/ml IL-3 (Peprotech, 213-13). On Day 4, 7, 13, 16, 19, the fresh media was added. MACS column magnetic separation was performed to isolate CD117(KIT)-positive cells using CD117 MicroBeads (Miltenyi Biotec, 130-091-224) and MACS buffer containing DPBS, 0.5% BSA, and 2 mM EDTA. Counting cell number was performed using Countess 2 (Thermo Fisher) automated cell counter. Bone marrow-derived macrophages were isolated and cultured as previously described^58^. Briefly, Bone marrow cells were cultured in DMEM supplemented with human macrophage colony-stimulating factor (PeproTec, 300-25), 10% FBS, 1% Pen/Strep, 2 mM L-glutamine, 1 mM sodium pyruvate.

### Immunofluorescence staining

Primary PC were mounted onto cytoslide using Cytopin 4 (Thermo Fisher). Cells were fixed with 4% paraformaldehyde (Thermo, 28908) for 10 min, washed with DPBS, permeabilized with a buffer containing DPBS and 0.1% Triton X-100, washed three times with a PBST washing buffer containing DPBS and 0.01% Tween 20 (Amresco, 0777), and subjected to blocking with a buffer containing PBST, 10% goat serum and 1% BSA for 1 h at 25 °C. After blocking, the cytoslide was incubated with a primary antibody [Phospho-JNK (Cell Signaling, 9255 S) or Phospho-MITF (Millipore Sigma, SAB4503940)] in a buffer containing PBST and 0.1% BSA for 18 h at 4 °C, washed three times with PBST, incubated with a secondary antibody [Alexa Fluor-488 Goat-anti-Rabbit IgG (Invitrogen, A11008) or Alexa Fluor-568 Goat-anti-Rabbit IgG (Invitrogen, A11011)] and Hoechst 33342 (Invitrogen, H3570) in a buffer containing PBST and 0.1% BSA for 1 h at 25 °C, and washed three times with PBST. After drying, the cytoslide was covered by coverslips with Flouromount-G mounting medium (SouthernBiotech, 0100-01) and stored at 4 °C for 18 h. Images were captured using a BZ-X710 series microscope (Keyence). Information on antibodies for immunofluorescence is presented in Supplementary Table 4.

### SCF intranasal challenge

12-week-old age- and sex-matched *Mcemp1*^+/+^ and *Mcemp1*^–/–^ mice were intraperitoneally (i.p.) anesthetized with 100 mg/kg ketamine HCl (Zoetis, NDC 54771-2013-1) and 10 mg/kg xylazine (PIVETAL, NDC 46066-750-02), and intranasally (i.n.) administered with 1 mg/kg SCF or 0.9% saline in 30–50 μl on days 1, 4, and 8. At 24 h after the last challenge, lungs were isolated for flow cytometry analysis.

### Ovalbumin (OVA)-induced chronic asthma model

This animal model has been previously described^34^. Briefly, 6–8-week-old age- and sex-matched *Mcemp1*^+/+^ and *Mcemp1*^–/–^ mice were i.p. immunized with 50 μg OVA (Worthington, LS003054) in 100 μl saline on days 1, 4, and 7. Starting on day 12, mice were i.p. anesthetized with 100 mg/kg ketamine HCl and 10 mg/kg xylazine, and i.n. challenged with 20 μg OVA in 30-50 μl saline weekly for a total of 8 weeks. Control mice received 0.9 % saline alone for i.p. injections and i.n. instillations. At 24 h after the last challenge, lung imaging was acquired using 4Dx scanner (4DMedical). Airway responses to aerosolized methacholine were measured using Flexivent system (SCIREQ)^59^. Mice were sacrificed and bronchoalveolar lavage (BAL) fluid was recovered by 700 μl of saline with 5% FBS and examined for cell numbers and types. BAL cells were counted in hematocytometer and differential leukocyte counts were performed visually in Eosin Y/Methylene Blue-stained cytospin preparations. Blood was obtained by cardiac puncture. BAL fluid (85 μl) and plasma (50 μl) were subjected to mouse MAP 4.1 Luminex platform multiplex analysis (Ampersand biosciences). The lung was perfused and isolated for histology, flow cytometry analysis, and RNA sequencing.

### Mast cell engraftment

Bone marrow cells from *Mcemp1*^+/+^ mice were culture with 30 ng/ml SCF and 30 ng/ml IL-3 for 4 weeks to generate cell populations that contained more than 95% immature mast cells. 10^7^ bone marrow-derived mast cells were intravenously injected into 10-week-old recipient mouse via the tail vein. At 12 weeks later, recipients were subjected to OVA-induced chronic asthma model.

### 4DMedical Imaging

The NHD Preclinical Scanner is an X-ray device, optimized for performing 4D CT of mouse lungs, which in conjunction with 4DMedical data processing delivers regional measurements of lung function. Each mouse was i.p. anesthetized with 100 mg/kg pentobarbital sodium (SAGENT Pharmaceuticals, NDC 25021-676-20) and after tracheotomy the trachea was cannulated using a 20ga ½ Luer Stub (Insteach Laboratories). The mouse was placed in an animal holder that was connected to the ventilator. A typical 4D CT scan took around 3-4 min and 4–10 scans/mouse were collected.

### Flexivent measurement of airway reactivity

Mice were i.p. anesthetized with 100 mg/kg pentobarbital sodium, tracheostomized, placed on a rodent ventilator flexivent (SCIREQ) set at a tidal volume of 10 ml/kg and at a rate of 200 breaths per min, and i.p. administered with 1 mg/kg with pancuronium bromide (Sigma, P1918-50MG). Mice were given a total of 2–4 serially increasing doses of methacholine (2.5–100 mg/ml, Sigma, A2251-100G) via a small volume nebulizer connected to the tracheostomy tube and ventilator. After each dose, the change in lung resistance was recorded on a computer.

### Histology

Tissues were fixed in 10% formaldehyde (Sigma, HT501128) and embedded in paraffin. Immunohistochemical staining was performed with MCEMP1 antibody (1:500 dilution) and counterstained with hematoxylin. For immunofluorescence staining, tissues were fixed in 4% paraformaldehyde, cryoprotected by 30% sucrose (JT Baker, 4072-05) in PBS until tissue sank, and cryopreserved in Tissue Freezing Medium (Electron Microscopy Sciences, 72592). Immunofluorescence staining was performed with MCEMP1 antibody (1:1000 dilution) and Alexa Fluor-568 Goat-anti-Rabbit IgG (Invitrogen, A11011). Mast cells were stained by 0.5% Toluidine blue and counterstain by 0.01% eosin. Number of toluidine blue positive cells was counted by Image J. Images were captured using a BZ-X710 series microscope (Keyence). Paraffin embedding, sectioning (7 μm), hematoxylin-eosin staining, Luna’s Toluidine blue staining, and Masson’s Trichrome staining were performed by the USC Immunohistochemistry Core facility and the Cleveland Clinic Imaging Histology Core facility.

### Flow cytometry

Surface staining of lung cells was performed with CD45-APC-Cy7 (BD, 557659), KIT-PE (BD, 553355), FcεRI-Alexa488 (BioLegend, 134330), Mouse Lineage Cocktail-APC (BD, 558074), Ly-6G-APC (BioLegend, 127614), Ly-6C-BV650 (BioLegend, 128049), CD11b-Alexa488 (BD, 557672). CD11c-BV421 (BD, 562782), Siglec-F-PE (BD, 552126), and CD16/CD32 (BD, 553142) antibodies. Blue Dead Cell Stain (Thermo Fisher, L34962) was used for fixable live/dead staining as per manufacturer instructions. Information on antibodies for flow cytometry is provided in Supplementary Table 5. FACS buffer contains DPBS, 5% FBS, and 0.02% sodium azide. Brilliant stain buffer plus (BD Biosciences, 566385) was added when using two or more BD Horizon Brilliant antibodies. Lungs were mechanically dispersed using scissors and enzymatically digested using 1 ml of 1.67 mg/ml collagenase IV (Worthington, LS004210) plus 10 μl of 10 mg/ml DNase Type IV (Sigma, D5025) per lobe for 1 h at 37 °C with shaking. After filtering through 40 μm strainer and RBC lysis (BioLegend, 420301), cells were incubated with Fc block in 50 μl FACS buffer for 10 min at 4 °C, then incubated with antibody cocktail of interest in total 100 μl FACS buffer for 30 min at 4 °C, washed with FACS buffer, and fixed with 4% paraformaldehyde for 15 min at 4 °C. CountBright absolute counting beads (Thermo Fisher, C36950) were used for calculating absolute cell numbers. UltraComp eBeads Plus compensation beads (Thermo Fisher, 01-3333-42) were used for compensation or unmixing. FACS acquisition was performed on BD FACS Celesta (BD Biosciences) and Sony ID7000 (Sony). Sony ID7000 acquisition was performed by the Cleveland Clinic LRI FlowCore. All flow cytometry data were analyzed using FlowJo v10.0 software.

### RNA-seq

The lung was lysed using TissueLyser II (Qiagen) for 30 frequency/s and 30 s. RNA from PCMC and lung was extracted using RNeasy kit according to the manufacturer’s protocol. Total RNA concentration was measured using a NanoDrop and processed into RNA sequencing libraries with the KAPA mRNA Hyperprep kit (Roche, 08098123702). Library quality control was tested using an Agilent TapeStation 4200 and sequencing was performed on an Illumina NextSeq 500 (PCMC) or NovaSeq 6000 (lung). Sequencing was handled by the University of Southern California Molecular Genomics Core and the Cleveland Clinic Genomics Core. Subsequent demultiplexed fastq files were analyzed with Partek Flow software, v10.0. Further analysis was performed using R (Core Team, 2014) and figures were produced with the ggplot2 package (Wickham, 2009). Pathway analysis was performed using Ingenuity Pathway Analysis (QIAGEN, version 01-20-04).

### Statistics and reproducibility

All experiments were independently repeated at least twice with similar results. Results are presented as mean±s.e.m., violin plot with lines at median and quartiles, or min to max of box and whiskers. For data analysis, one-way ANOVA with Tukey’s multiple comparisons; two-way ANOVA with Tukey’s or Sidak’s multiple comparisons; two-tailed unpaired Student’s *t*-test for two-component comparisons determined by GraphPad Prism 9.0. For RNA seq data analysis, right-tailed Fisher’s Exact Test determined by Ingenuity Pathway Analysis version 01-20-04; *p*-values in volcano plots determined by Gene Specific Analysis in Partek Flow software, v10.0.

### Reporting summary

Further information on research design is available in the Nature Portfolio Reporting Summary linked to this article.

## Supplementary information

Supplementary Information

Reporting Summary

## Data Availability

RNA-seq data generated in this study have been deposited in the GEO database under accession code GSE197042 Subseries, GSE197040 (lung) and GSE197041 (PCMC). All other data are available in the article and its Supplementary files or from the corresponding author upon request. Source data are provided with this paper.

## Source data

Source Data
