# Supplementary material for: Lung-specific MCEMP1 functions as an adaptor for KIT to promote SCF-mediated mast cell proliferation
Source: Nat Commun. 2023 Apr 11;14:2045. doi: 10.1038/s41467-023-37873-3 (PMC10090139; doi:10.1038/s41467-023-37873-3)

Supplementary Table 1: Mass spectrometry peptide sequence

SOS1

MQAQQLPYEF FSEENAPKWR GLLVPALKKV QGQVHPTLES NDDALQYVEE LILQLLNMLC QAQPRSASDV
EERVQKSFPH PIDK**WAIADA QSAIEK**RKRR **NPLSLPVEK**I HPLLKEVLGY KIDHQVSVYI VAVLEYISAD
ILKLVGNYVR NIRHYEITKQ DIKVAMCADK VLMDMFHQDV EDINILSLTD EEPSTSGEQT YYDLVKAFMA
EIRQYIRELN LIIK**VFREPF VSNSKLFSAN DVENIFSRIV DIHELSVK**LL GHIEDTVEMT DEGSPHPLVG
SCFEDLAEEL AFDPYESYAR DILRPGFHDR FLSQLSKPGA ALYLQSIGEG FK**EAVQYVLP R**LLLAPVYHC
LHYFELLKQL EEKSEDQEDK ECLK**QAITAL LNVQSGMEK**I CSKSLAKRRL SESACRFYSQ QMKGKQLAIK
KMNEIQK**NID GWEGK**DIGQC CNEFIMEGTL TRVGAKHERH IFLFDGLMIC CKSNHGQPR**L PGASNAEYR**L
KEKFFMR**KVQ INDKDDTNEY KHAFEIILKD ENSVIFSAK**S AEEKNNWMAA LISLQYRSTL ER**MLDVTMLQ
EEKEEQMRLP SADVYRFAEP DSEENIIFEE NMQPK**AGIPI IKAGTVIKLI ER**LTYHMYAD PNFVRTFLTT
YR**SFCKPQEL LSLIIER**FEI PEPEPTEADR IAIENGDQPL SAELKR**FRK**E YIQPVQLR**VL NVCRHWVEHH
FYDFERDAYL LQR**MEEFIGT VR**GKAMKK**WV ESITK**IIQRK KIARDNGPGH NITFQSSPPT VEWHISRPGH
IETFDLLTLH PIEIAR**QLTL LESDLYRAVQ PSELVGSVWT KEDKEINSPN LLK**MIR**HTTN LTLWFEK**CIV
ETENLEERVA VVSRIIEILQ VFQELNNFNG VLEVVSAMNS SPVYR**LDHTF EQIPSR**QKK**I LEEAHELSED
HYK**KYLAKLR SINPPCVPFF GIYLTNILK**T EEGNPEVLKR** HGKELINFSK RRKVAEITGE IQQYQNQPYC
LRVESDIKR**F FENLNPMGNS MEKEFTDYLF NK**SLEIEPRN PKPLPRFPKK YSYPLKSPGV RPSNPRPGTM
R**HPTPLQQEP R**KISYSR**IPE SETESTASAP NSPR**TPLTPP PASGASSTTD VCSVFDSDHS SPFHSSNDTV
FIQVTLPHGP R**SASVSSISL TKGTDEVPVP PPVPPR**RRPE SAPAESSPSK IMSKHLDSPP AIPPRQPTSK
AYSPRYSISD RTSISDPPES PPLLPPREPV R**TPDVFSSSP LHLQPPPLGK** KSDHGNAFFP NSPSPFTPPP
PQTPSPHGTR R**HLPSPPLTQ EVDLHSIAGP PVPPR**QSTSQ HIPKLPPKTY KREHTHPSMH R**DGPPLLENA HSS**

SOS2

MQQAPQPYEF FSEENSPKWR GLLVSALRKV QEQVHPTLSA NEESLYYIEE LIFQLLNKLC MAQPRTVQDV EERVQKTFPH
PIDKWAIADA QSAIEKRKRR NPLLLPVDKI HPSLKEVLGY KVDYHVSLYI VAVLEYISAD ILK**LAGNYVF NIRHYEISQQ
DIK**VSMCADK VLMDMFDQDD IGLVSLCEDE PSSSGELNYY DLVRTEIAEE RQYLRELNMI IKVFR**EAFLS DR**KLFKPSDI
EKIFSNISDI HELTVKLLGL IEDTVEMTDE SSPHPLAGSC FEDLAEEQAF DPYETLSQDI LSPEFHEHFN KLMARPAVAL
HFQSIADGFK EAVRYVLPRL MLVPVYHCWH YFELLKQLKA CSEEQEDREC LNQAITALMN LQGSMDRIYK QYSPRRRPGD
PVCPFYSHQL RSKHLAIKKM NEIQKNIDGW EGKDIGQCCN EFIMEGPLTR IGAKHERHIF LFDGLMISCK PNHGQTR**LPG
YSSAEYR**LKE KFVMRKIQIC DKEDTCEHK**H AFELVSKDEN SIIFAAK**SAE EKNNWMAALI SLHYRSTLDR **MLDSVLLKEE
NEQPLR**LPSP EVYRFVVK**DS EENIVFEDNL QSR**SGIPIIK GGTVVKLIER LTYHMYADPN FVRTFLTTYR SFCKPQELLS
LLIER**FEIPE PEPTDADK**LA IEK**GEQPISA DLK**RFRK**EYV QPVQLR**ILNV FRHWVEHHFY DFERDLELLE R**LESFISSVR**
GKAMKKWVES IAKIIRRKKQ AQANGVSHNI TFESPPPPIE WHISKPGQFE TFDLMTLHPI EIARQLTLLE SDLYRKVQPS
ELVGSVWTKE DKEINSPNLL KMIRHTTNLT LWFEKCIVEA ENFEERVAVL SRIIEILQVF QDLNNFNGVL EIVSAVNSVS
VYRLDHTFEA LQERKR**KILD EAVELSQDHF K**KYLVKLKSI NPPCVPFFGI YLTNILK**TEE GNNDFLK**KK**G KDLINFSK**RR
KVAEITGEIQ QYQNQPYCLR IEPDMRR**FFE NLNPMGSASE K**EFTDYLFNK SLEIEPRNCK QPPRFPRKST FSLKSPGIRP
NTGR**HGSTSG TLR**GHPTPLE REPCKISFSR IAETELESTV SAPTSPNTPS TPPVSASSDL SVFLDVDLNS SCGSNSIFAP
VLLPHSKSFF SSCGSLHK**LS EEPLIPPPLP PR**KKFDHDAS NSKGNMK**SDD DPPAIPPR**QP PPPKVKPR**VP VPTGAFDGPL
HSPPPPPPR**D PLPDTPPPVP LRPPEHFINC PFNLQPPPLG HLHRDSDWLR DISTCPNSPS TPPSTPSPRV PRRCYVLSSS
QNNLAHPPAP PVPPRQNSSP HLPKLPPKTY KRELSHPPLY R**LPLLENAET PQ**

Grb2

MEAIAKYDFK **ATADDELSFK** RGDILKVLNE ECDQNWYKAE LNGKDGFIPK NYIEMKPHPW FFGKIPR**AKA EEMLSK**QR**HD GAFLIRESES APGDFSLSVK
FGNDVQHFK**V LRDGAGKYFL WVVKFNSLNE LVDYHRSTSV SRNQQIFLRD IEQMPQQPTY VQALFDFDPQ EDGELGFRRG DFIHVMDNSD PNWWKGACHG
QTGMFPRNYV TPVNRNV

Supplementary Table 2: List of antibodies used for immunoblotting

Antibody	Catalog	Dilution	Time
MCEMP1	N/A	1:1000 in 5 % milk in TBST	18 h, 4°C
Phospho-KIT	Cell Signaling, 3391S	1:1000 in 5% BSA in TBST	18 h, 4°C
KIT	Cell Signaling, 3074S	1:2000 in 5% BSA in TBST	18 h, 4°C
SOS1	Cell Signaling, 12409S	1:1000 in 5% BSA in TBST	18 h, 4°C
Grb2	Santa Cruz, C-23	1:1000 in 5% BSA in TBST	18 h, 4°C
4G10	EMD Millipore, 05-321	1:1000 in 5% BSA in TBST	18 h, 4°C
4G10-HRP	EMD Millipore, 16-105	1:1000 in 5% BSA in TBST	18 h, 4°C
P-Tyr-1000	Cell Signaling, 8952S	1:1000 in 5% BSA in TBST	18 h, 4°C
Phospho-IκBα	Cell Signaling, 9241S	1:1000 in 5% BSA in TBST	18 h, 4°C
IκBα	Cell Signaling, 4814S	1:1000 in 5% BSA in TBST	18 h, 4°C
Phospho-MEK1/2	Cell Signaling, 9154S	1:1000 in 5% BSA in TBST	18 h, 4°C
MEK1/2	Cell Signaling, 9122S	1:1000 in 5% BSA in TBST	18 h, 4°C
Phospho-p38	Cell Signaling, 9211S	1:1000 in 5% BSA in TBST	18 h, 4°C
p38	Cell Signaling, 9212S	1:1000 in 5% BSA in TBST	18 h, 4°C
Phospho-JNK	Cell Signaling, 9251S	1:1000 in 5% BSA in TBST	18 h, 4°C
JNK	Cell Signaling, 4668S	1:1000 in 5% BSA in TBST	18 h, 4°C
Phospho-ERK	Cell Signaling, 4370S	1:1000 in 5% BSA in TBST	18 h, 4°C
ERK	Cell Signaling, 4695S or 9101S	1:1000 in 5% BSA in TBST	18 h, 4°C
GST	Santa Cruz, B-14	1:1000 in 5 % milk in TBST	18 h, 4°C
Actin	Santa Cruz, C4	1:1000 in 5 % milk in TBST	18 h, 4°C
Tubulin	Santa Cruz, B7	1:1000 in 5 % milk in TBST	18 h, 4°C
rabbit-Flag	Sigma, F7425	1:1000 in 5 % milk in TBST	18 h, 4°C
mouse-Flag	Sigma, F1804	1:1000 in 5 % milk in TBST	18 h, 4°C
rabbit-HA	Covance, PRB-101P	1:1000 in 5 % milk in TBST	18 h, 4°C
mouse-HA	BioLegend, 16B12	1:1000 in 5 % milk in TBST	18 h, 4°C
mouse Flag-HRP	abcam ab49763, M2	1:4000 in 5 % milk in TBST	1 h, 23°C
mouse HA-HRP	BioLegend 901519, 16B12	1:4000 in 5 % milk in TBST	1 h, 23°C

mouse V5-HRP	Thermo R961-25	1:5000 in 5 % milk in TBST	1 h, 23°C
mouse IgG-HRP	Cell Signaling, 7076	1:1000 in 5 % milk in TBST	1 h, 23°C
rabbit IgG-HRP	Cell Signaling, 7074	1:1000 in 5 % milk in TBST	1 h, 23°C

Supplementary Table 3: List of qRT-PCR primers

Gene	Sense primer (5'-3')	Antisense primer (5'-3')
Mouse *Gapdh*	GGTCCTCAGTGTAGCCCAAG	AATGTGTCCGTCGTGGATCT
Mouse *Il4*	AGATGGATGTGCCAAACGTCC	AATATGCGAAGCACCTTGGAA
Mouse *Il5*	TCACCGAGCTCTGTTGACAA	CCACACTTCTCTTTTTGGCG
Mouse *Il6*	TATAATCAGGAAATTTGCCTA	GTTAGGAGAGCATTGGAAAT
Mouse *Il10*	GCCAAGCCTTATCGGAAATG	AAATCACTCTTCACCTGCTCC
Mouse *Il13*	TGCGGTTACAGAGGCCATGCA	TGAGGAGCTGAGCAACATCAC
Mouse *Tnf*	CTTCTGTCTACTGAACTTCGGG	TGATCTGAGTGTGAGGGTCTG
Mouse *Ifng*	GCTTTGCAGCTCTTCCTCAT	GTCACCATCCTTTTGCCAGT
Mouse *Ccl2*	CATCCACGTGTTGGCTCA	GATCATCTTGCTGGTGAATGAGT
Mouse *Ccl4*	GCTCCAAGCCAGCTGTGGTA	CGCTGGAGCTGCTCAGTTC
Mouse *Ccl11*	CAGATGCACCCTGAAAGCCATA	TGCTTTGTGGCATCCTGGAC
Mouse *Ccr5*	CGTTCCCCCTACAAGAGACT	ACCCACAAAACCAAAGATGA
Mouse *Cxcl10*	TGCTGGGTCTGAGTGGGACT	CCCTATGGCCCTCATTCTCAC

Supplementary Table 4: List of antibodies used for immunofluorescence

Antibody	Catalog	Dilution	Time
Phospho-JNK	Cell Signaling, 9255S	1:200 in 0.1% BSA in PBST	18 h, 4°C
Phospho-MITF	Millipore Sigma, SAB4503940	1:500 in 0.1% BSA in PBST	18 h, 4°C
Alexa Fluor-488 Goat-anti-Rabbit IgG	Invitrogen, A11008	1:500 in 0.1% BSA in PBST	1 h, 23°C
Alexa Fluor-568 Goat-anti-Rabbit IgG	Invitrogen, A11011	1:500 in 0.1% BSA in PBST	1 h, 23°C
Hoechst 33342	Invitrogen, H3570	1:5000	N/A

Supplementary Table 5: List of antibodies used for flow cytometry

Antibody	Catalog	Dilution
CD45-APC-Cy7	clone 30-F11; BD, 557659	1:100
KIT(CD117)-PE	clone 2B8; BD, 553355	1:200
FcεRI-Alexa488	clone MAR-1, Biolegend, 134330	1:200
Mouse Lineage Cocktail-APC	BD, 558074	10 µl / $5x10^5$ cells
Ly-6G-APC	clone 1A8; Biolegend, 127614	1:200
Ly-6C-BV650	clone HK1.4; Biolegend, 128049	1:200
CD11b-Alexa488	clone M1/70; BD, 557672	1:200
CD11c-BV421	clone HL3; BD, 562782	1:200
Siglec-F-PE	clone E50-2440; BD, 552126	1:200
LIVE/DEAD Fixable Blue	Thermo Fisher, L34962	1 µl / million cells
CD16/CD32	BD, 553142	1 µg (2 µl) / million cells

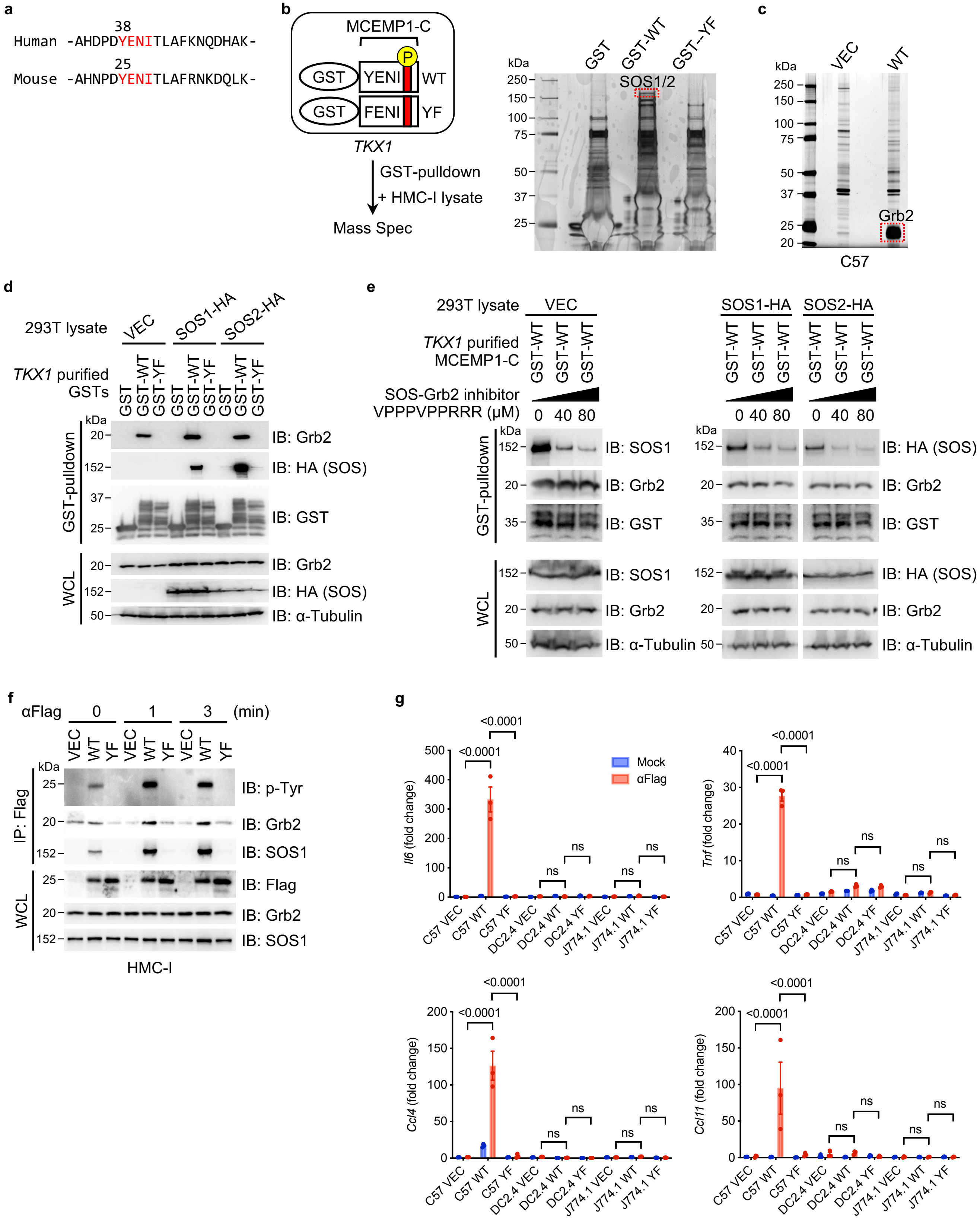
a
Human -AHDPDYENITLAFKNQDHAK-
38
Mouse -AHNPDYENITLAFRNKDQLK-
25
b
MCEMP1-C
GST YENI WT
GST FENI YF
P
TKX1
GST-pulldown
+ HMC-I lysate
Mass Spec
kDa
GST GST-WT GST--YF
SOS1/2
c
kDa
VEC WT
Grb2
C57
d
293T lysate
VEC SOS1-HA SOS2-HA
TKX1 purified GSTs
GST GST-WT GST-YF
GST-pulldown
WCL
IB: Grb2
IB: HA (SOS)
IB: GST
IB: α-Tubulin
e
293T lysate
VEC SOS1-HA SOS2-HA
TKX1 purified MCEMP1-C
SOS-Grb2 inhibitor VPPPVPPRRR (μM)
0 40 80
IB: SOS1
IB: Grb2
IB: GST
IB: HA (SOS)
IB: α-Tubulin
f
αFlag 0 1 3 (min)
VEC WT YF
IP: Flag
WCL
IB: p-Tyr
IB: Grb2
IB: SOS1
IB: Flag
HMC-I
g
Mock
αFlag
Il6 (fold change)
Tnf (fold change)
Ccl4 (fold change)
Ccl11 (fold change)
<0.0001
ns
C57 VEC
C57 WT
C57 YF
DC2.4 VEC
DC2.4 WT
DC2.4 YF
J774.1 VEC
J774.1 WT
J774.1 YF

Supplementary Figure 1 MCEMP1 interacts with Grb2-SOS signal complex and induces ITAM-dependent signal transduction.

a, ITAM motif sequence alignment of MCEMP1 from human and mouse. The conserved YENI residue is shown in red font. **b**, Schematics of *TKX1* expression system and identification of SOS1/2 as the phosphorylated MCEMP1 interacting proteins. *E. coli TKX1* strain contains elk tyrosine kinase which has broad specificity and efficiently phosphorylates mammalian proteins. *TKX1* strain was transformed with GST-tagged plasmids encoding human MCEMP1 cytoplasmic domain (MCEMP1-C) either wild-type (GST-WT) or tyrosine to phenylalanine mutant (GST-YF). Purified GST-tagged MCEMP1 proteins were incubated with HMC-1 cell lysates. MCEMP1 protein complexes were enriched by GST-pulldown and visualized on silver stained-SDS-PAGE. Mass spectrometry identified SOS1/2 from 150 kDa size band in GST-WT lane. **c**, Identification of Grb2 as the phosphorylated MCEMP1 interacting protein. MCEMP1 proteins were purified from C57 cells expressing vector control (VEC) or wild-type MCEMP1 (WT) by immunoprecipitation (IP) with anti-Flag antibody. MCEMP1 protein complexes were visualized on silver stained-SDS-PAGE. Mass spectrometry identified Grb2 from 25 kDa size band of WT MCEMP1. **d**, GST-pulldown assay of MCEMP1 interaction with Grb2-SOS1/2 signal complex in an ITAM dependent manner. GST-pulldown elutes and whole cell lysates (WCL) were analyzed by immunoblotting (IB) with the indicated antibodies. **e**, Effect of SOS SH3 domain inhibitor on MCEMP1 interaction with SOS in 293T cells. **f**, MCEMP1 tyrosine phosphorylation and its interaction with Grb2 and SOS1 in HMC-1 cells. HMC-1 cells expressing VEC, WT MCEMP1, or YF mutant MCEMP1 were treated with αFlag for the indicated time and cell lysates were IP with anti-Flag antibody. Immunoprecipitates and WCL were analyzed by IB with the indicated antibodies. **g**, Gene expressions of *Il6*, *Tnf*, *ccl4*, and *ccl11* in C57, DC2.4, or J774.1 cells after αFlag treatment. Data are representative of at least two independent experiments in **b**-**f**. Data are presented by mean ± s.e.m. and *p*-values were determined by two-way ANOVA with Sidak's multiple comparison in **g** (n=3). ns, not significant.

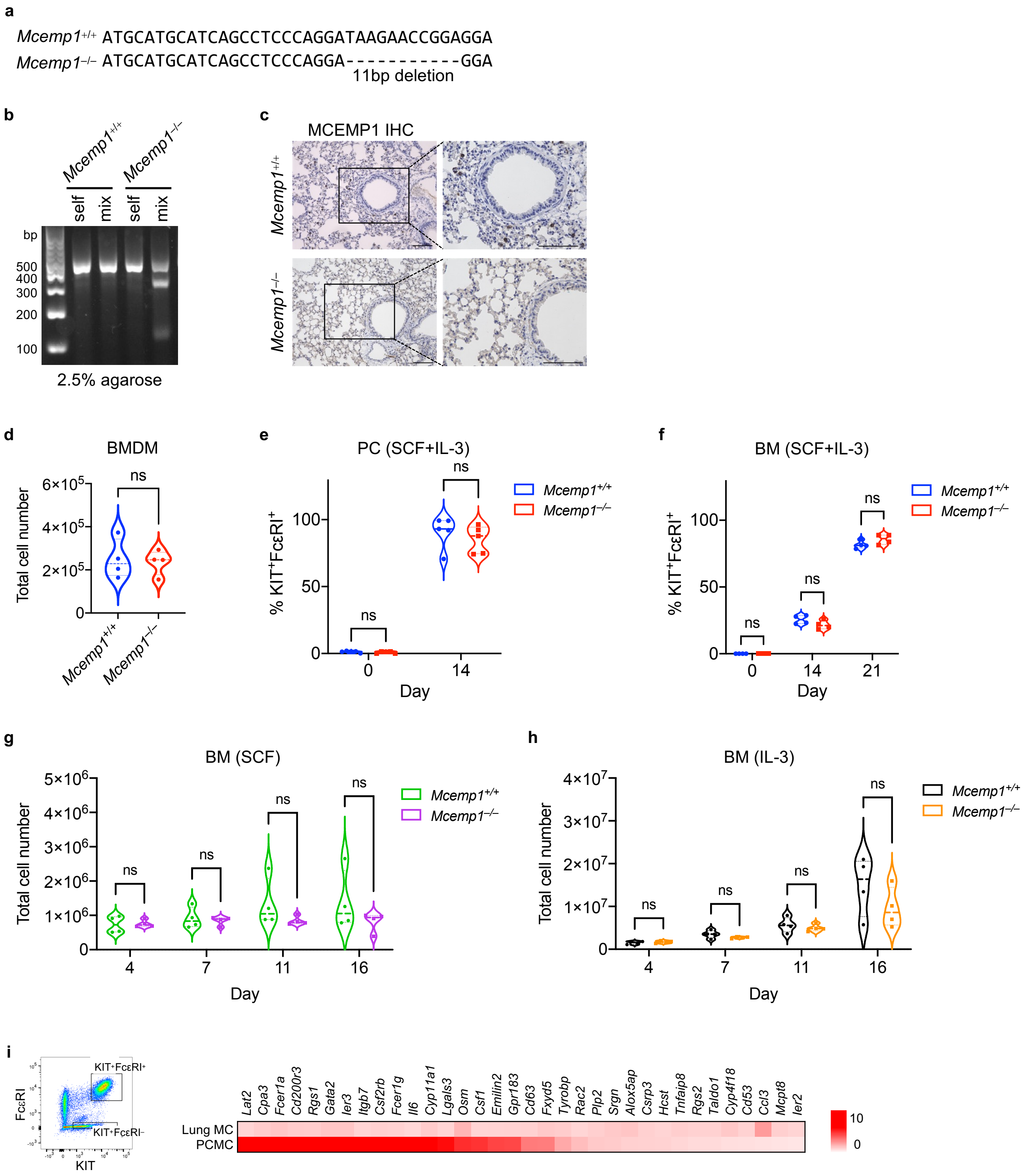
a
Mcemp1+/+ ATGCATGCATCAGCCTCCCAGGATAAGAACCGGAGGA
Mcemp1–/– ATGCATGCATCAGCCTCCCAGGA-----------GGA
11bp deletion
b
Mcemp1+/+
Mcemp1–/–
self
mix
self
mix
bp
500
400
300
200
100
2.5% agarose
c
MCEMP1 IHC
Mcemp1+/+
Mcemp1–/–
d
BMDM
Total cell number
6×10^5
4×10^5
2×10^5
0
ns
Mcemp1+/+
Mcemp1–/–
e
PC (SCF+IL-3)
% KIT+FcεRI+
100
50
0
ns
ns
0
14
Day
Mcemp1+/+
Mcemp1–/–
f
BM (SCF+IL-3)
% KIT+FcεRI+
100
50
0
ns
ns
ns
0
14
21
Day
Mcemp1+/+
Mcemp1–/–
g
BM (SCF)
Total cell number
5×10^6
4×10^6
3×10^6
2×10^6
1×10^6
0
ns
ns
ns
ns
4
7
11
16
Day
Mcemp1+/+
Mcemp1–/–
h
BM (IL-3)
Total cell number
4×10^7
3×10^7
2×10^7
1×10^7
0
ns
ns
ns
ns
4
7
11
16
Day
Mcemp1+/+
Mcemp1–/–
i
KIT+FcεRI+
KIT+FcεRI–
FcεRI
KIT
Lat2
Cpa3
Fcer1a
Cd200r3
Rgs1
Gata2
Ier3
Itgb7
Csf2rb
Fcer1g
Il6
Cyp11a1
Lgals3
Osm
Csf1
Emilin2
Gpr183
Cd63
Fxyd5
Tyrobp
Rac2
Plp2
Srgn
Alox5ap
Csrp3
Hcst
Tnfaip8
Rgs2
Taldo1
Cyp4f18
Cd53
Ccl3
Mcpt8
Ier2
Lung MC
PCMC
10
0

Supplementary Figure 2 Generation of *Mcemp1*$^{-/-}$ mice.

a, Genomic DNA sequence of the first coding region of *Mcemp1* from *Mcemp1*$^{+/+}$ or *Mcemp1*$^{-/-}$ littermate mice. 11 base pairs out-of-frame deletion was shown in *Mcemp1*$^{-/-}$ mice. **b**, Genotyping of *Mcemp1*$^{+/+}$ and *Mcemp1*$^{-/-}$ mice by T7 endonuclease I assay. bp, base pair. **c**, Loss of MCEMP1 protein expression in *Mcemp1*$^{-/-}$ mice. Immunohistochemistry staining for MCEMP1 expression in the lungs. Scale bar, 100 μm. **d**, Total cell numbers of *Mcemp1*$^{+/+}$ or *Mcemp1*$^{-/-}$ bone marrow-derived macrophages (BMDM) cultured with macrophage colony-stimulating factor (MCSF) for 7 days (n=4 per group). **e**,**f**, Percentages of KIT/FcεRI double-positive PC (e) or BM (f) from *Mcemp1*$^{+/+}$ or *Mcemp1*$^{-/-}$ mice upon SCF and IL-3 stimulation (n=4-5 per group). **g**,**h**, Absolute cell counts of *Mcemp1*$^{+/+}$ or *Mcemp1*$^{-/-}$ BM cultured with either SCF (g) or IL-3 (h) for the indicated days (n=4 per group). **i**, Representative flow cytometry plots illustrating the gating strategy to identify KIT and FcεRI double positive cells (KIT^{+}FcεRI^{+}) and KIT single positive cells (KIT^{+}FcεRI^{-}). Heatmaps of top DEGs of KIT^{+}FcεRI^{+} PCMC versus KIT^{+}FcεRI^{-} cells and lung mast cells (MC) versus other lung cells. Images are representative of two independent experiments in **b** and **c**. Data are presented as violin plots with lines at median and quartiles in **d**-**h**. *P*-values were determined by two-tailed unpaired *t*-test in **d** and by two-way ANOVA with Sidak's multiple comparison in **e**-**h**. ns, not significant.

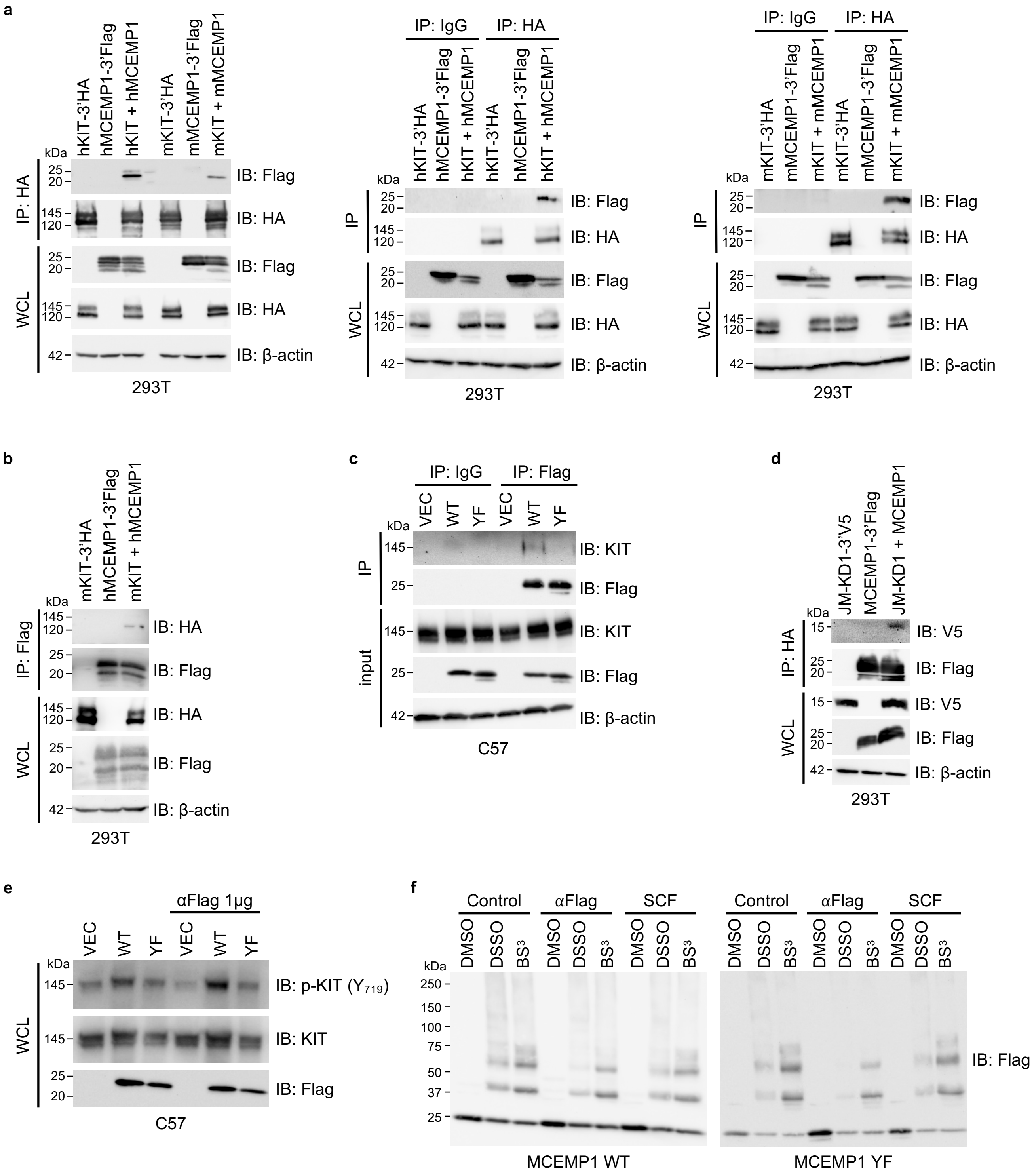
a
hKIT-3'HA
hMCEMP1-3'Flag
hKIT + hMCEMP1
mKIT-3'HA
mMCEMP1-3'Flag
mKIT + mMCEMP1
kDa
25
20
145
120
42
IP: HA
WCL
IB: Flag
IB: HA
IB: β-actin
293T
IP: IgG
IP: HA
hKIT-3'HA
hMCEMP1-3'Flag
hKIT + hMCEMP1
IP
WCL
IB: Flag
IB: HA
IB: β-actin
293T
IP: IgG
IP: HA
mKIT-3'HA
mMCEMP1-3'Flag
mKIT + mMCEMP1
IP
WCL
IB: Flag
IB: HA
IB: β-actin
293T
b
mKIT-3'HA
hMCEMP1-3'Flag
mKIT + hMCEMP1
IP: Flag
WCL
IB: HA
IB: Flag
IB: β-actin
293T
c
IP: IgG
IP: Flag
VEC
WT
YF
IP
input
IB: KIT
IB: Flag
IB: β-actin
C57
d
JM-KD1-3'V5
MCEMP1-3'Flag
JM-KD1 + MCEMP1
15
IP: HA
WCL
IB: V5
IB: Flag
IB: β-actin
293T
e
αFlag 1μg
VEC
WT
YF
WCL
IB: p-KIT (Y719)
IB: KIT
IB: Flag
C57
f
Control
αFlag
SCF
DMSO
DSSO
BS3
250
150
100
75
50
37
25
IB: Flag
MCEMP1 WT
MCEMP1 YF

Supplementary Figure 3 MCEMP1 enhances KIT autophosphorylation.

a, Co-immunoprecipitation assay for human and mouse MCEMP1 and KIT interaction in 293T cells. **b**, Co-immunoprecipitation assay of human MCEMP1 and mouse KIT in 293T cells. **c**, Co-immunoprecipitation assay of MCEMP1 and KIT in C57 cells expressing vector (VEC), wild-type MCEMP1 (WT), or YF mutant (YF). Cell lysates were immunoprecipitated with anti-Flag antibody or IgG control. **d**, Co-immunoprecipitation assay for MCEMP1 interaction with KIT truncation construct (JM-KD1) in 293T cells. **e**, Immunoblot analysis of KIT phosphorylation upon αFlag stimulation in C57 cells. **f**, MCEMP1 oligomers captured by disuccinimidyl suberate (DSS) or bis(sulfosuccinimidyl) suberate (BS^3) non-cleavable crosslinkers. C57 cells expressing MCEMP1 WT or YF mutant were stimulated with control, αFlag, or SCF and then subjected to 2.5 mM DSS or BS^3 crosslinking for 30 min. Crosslinked cells were used for immunoblotting with anti-Flag antibody. Data are representative of at least two independent experiments in **a**-**f**.

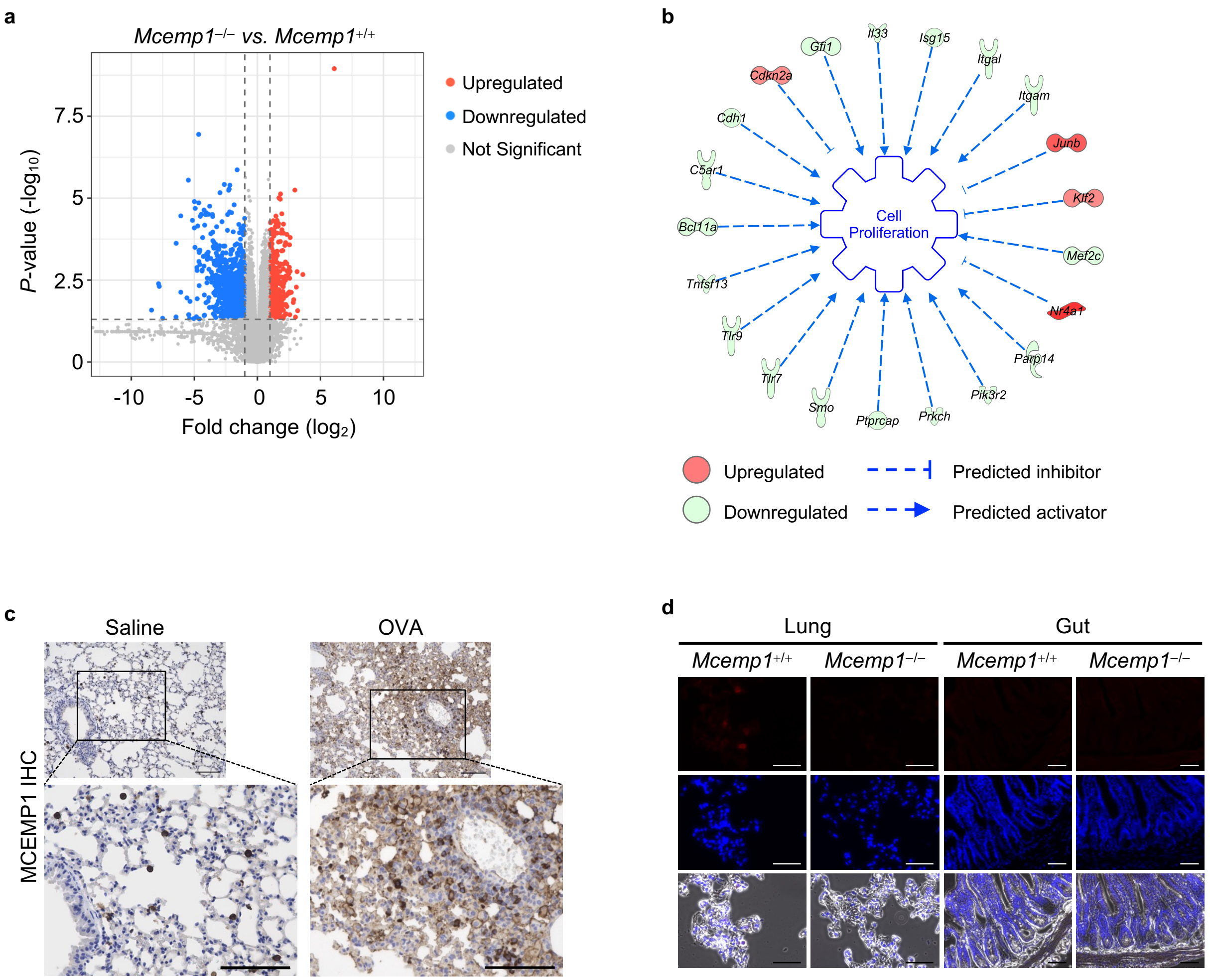

Supplementary Figure 4 Altered gene expressions in *Mcemp1*-deficient PCMC and MCEMP1 expression in the lungs during OVA stimulation.

a, RNA-seq volcano plot of DEGs from *Mcemp1*$^{-/-}$ PCMC versus *Mcemp1*$^{+/+}$ PCMC. The volcano plot is presented as fold change in gene expression ($\log_2$ Fold change) against significance of change ($-\log_{10}$ *P*-value). The significantly upregulated genes are indicated in red and downregulated gene are indicated in blue. The cutoff of significant DEGs is a |Fold change| > 2 and *P*-value < 0.05. *p*-values in b-d were determined by Gene Specific Analysis (GSA) in Partek Flow software. **b**, IPA network association of DEGs from *Mcemp1*$^{-/-}$ PCMC versus *Mcemp1*$^{+/+}$ PCMC. Downregulated and upregulated genes are shown in green and red, respectively. **c**, Immunohistochemistry staining for MCEMP1 expression in the lung challenged with OVA. Scale bar, 100 μm. **d**, Immunofluorescent staining of MCEMP1 expression in the lung and gut of *Mcemp1*$^{+/+}$ and *Mcemp1*$^{-/-}$ mice challenged with OVA. Anti-MCEMP1 antibody (red) or DAPI (blue). Scale bar, 50 μm. Data are representative of two independent experiments in **c**,**d**.

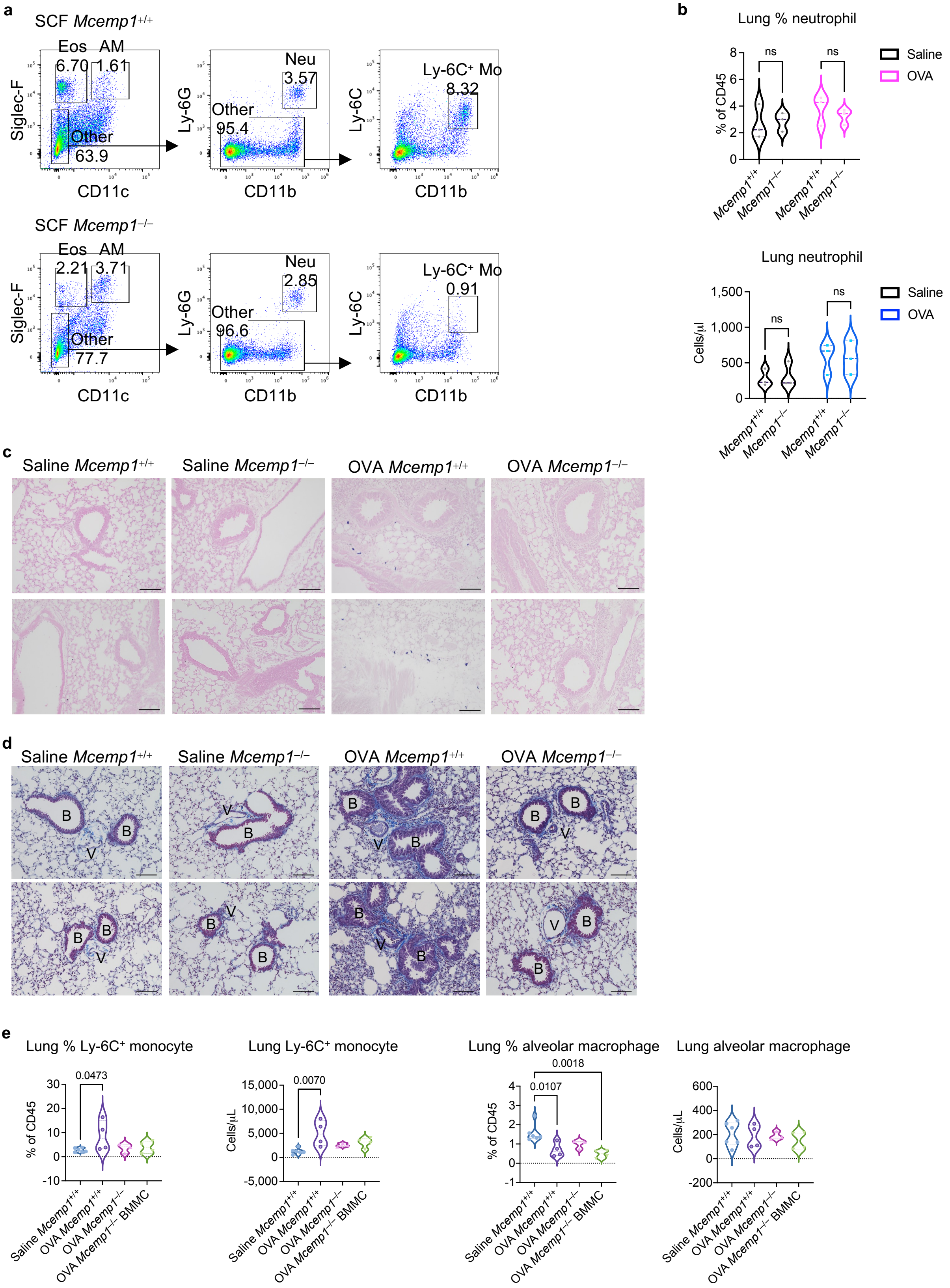
a
SCF Mcemp1+/+
Eos AM
6.70 1.61
Other
63.9
Siglec-F
CD11c
Neu
3.57
Other
95.4
Ly-6G
CD11b
Ly-6C+ Mo
8.32
Ly-6C
CD11b
SCF Mcemp1–/–
Eos AM
2.21 3.71
Other
77.7
Siglec-F
CD11c
Neu
2.85
Other
96.6
Ly-6G
CD11b
Ly-6C+ Mo
0.91
Ly-6C
CD11b
b
Lung % neutrophil
% of CD45
ns
ns
Saline
OVA
Mcemp1+/+
Mcemp1–/–
Mcemp1+/+
Mcemp1–/–
Lung neutrophil
Cells/µl
ns
ns
Saline
OVA
Mcemp1+/+
Mcemp1–/–
Mcemp1+/+
Mcemp1–/–
c
Saline Mcemp1+/+
Saline Mcemp1–/–
OVA Mcemp1+/+
OVA Mcemp1–/–
d
Saline Mcemp1+/+
Saline Mcemp1–/–
OVA Mcemp1+/+
OVA Mcemp1–/–
B
V
e
Lung % Ly-6C+ monocyte
0.0473
% of CD45
Lung Ly-6C+ monocyte
0.0070
Cells/µL
Lung % alveolar macrophage
0.0107
0.0018
% of CD45
Lung alveolar macrophage
Cells/µL
Saline Mcemp1+/+
OVA Mcemp1+/+
OVA Mcemp1–/–
OVA Mcemp1–/– BMMC

Supplementary Figure 5 MCEMP1 exacerbates airway inflammation in OVA-induced chronic asthma model.

a, Representative flow cytometry plots illustrating the gating strategy to identify eosinophils (Eos), alveolar macrophages (AM), neutrophils (Neu), and Ly-6C-positive inflammatory monocytes (Mo). **b**, Percentages and absolute numbers of neutrophils in the lung were determined by flow cytometry (n=3 per group). **c**, Representative images of toluidine blue staining for mast cell counts in lung section. Eosin was used for counterstaining. Scale bar, 100 μm. **d**, Representative images of Masson's trichrome staining for collagen deposition. Collagen fibers were stained blue in the perivascular and parabronchial tissues. Scale bar, 100 μm. **e**, Percentages and absolute numbers of Ly-6C+ inflammatory monocytes and alveolar macrophages in the lungs were determined by flow cytometry (n=6 for saline group. n=3-4 for OVA group). Data are representative of two independent experiments in **c** and **d**. Data are presented as violin plots with lines at median and quartiles and *p*-values were determined by two-way ANOVA with Sidak's multiple comparison in **b** or by one-way ANOVA with Tukey's multiple comparison in **e**. ns, not significant.

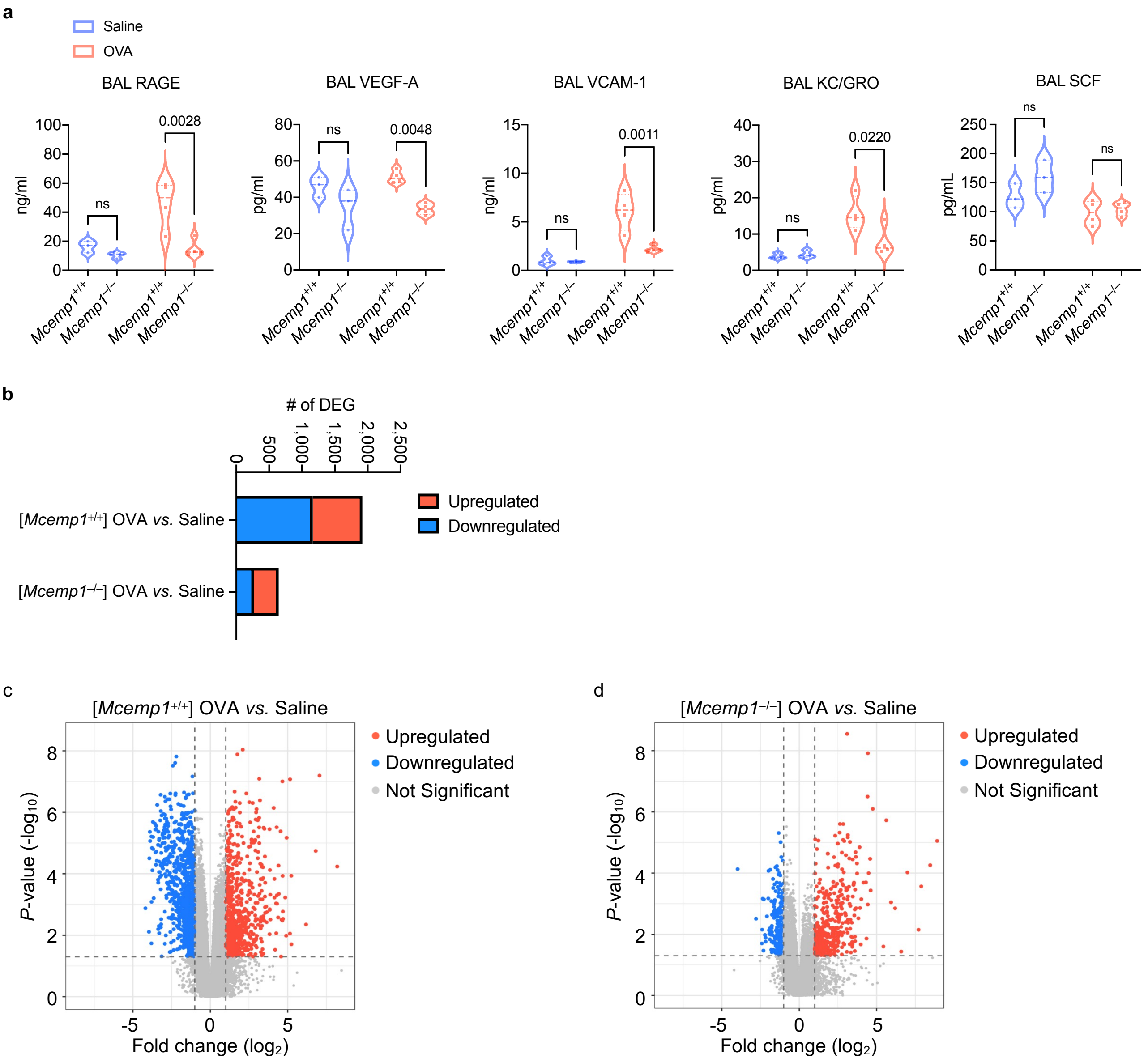

Supplementary Figure 6 Altered gene expressions in *Mcemp1*-deficient mice.

a, Multiplex analysis of cytokines and growth factors in the BAL (n=3 for saline group, n=4 for OVA group). Data are presented as violin plots with lines at median and quartiles and *p*-values were determined by two-way ANOVA with Sidak's multiple comparison. ns, not significant. **b**, The total number of significant DEGs from RNA-seq. The cutoff of significant DEGs is a |Fold change| > 2 and *P*-value < 0.05. **c**,**d**, The volcano plots of DEGs from OVA-challenged versus saline-treated *Mcemp1*$^{+/+}$ mice (c) or OVA-challenged versus saline-treated *Mcemp1*$^{–/–}$ mice (d). The volcano plot is presented as fold change in gene expression ($\log_2$ Fold change) against significance of change ($-\log_{10}$ *P*-value). The significantly upregulated genes are denoted in red and downregulated gene are indicated in blue. *p*-values were determined by Gene Specific Analysis (GSA) in Partek Flow software in **b**-**d**.

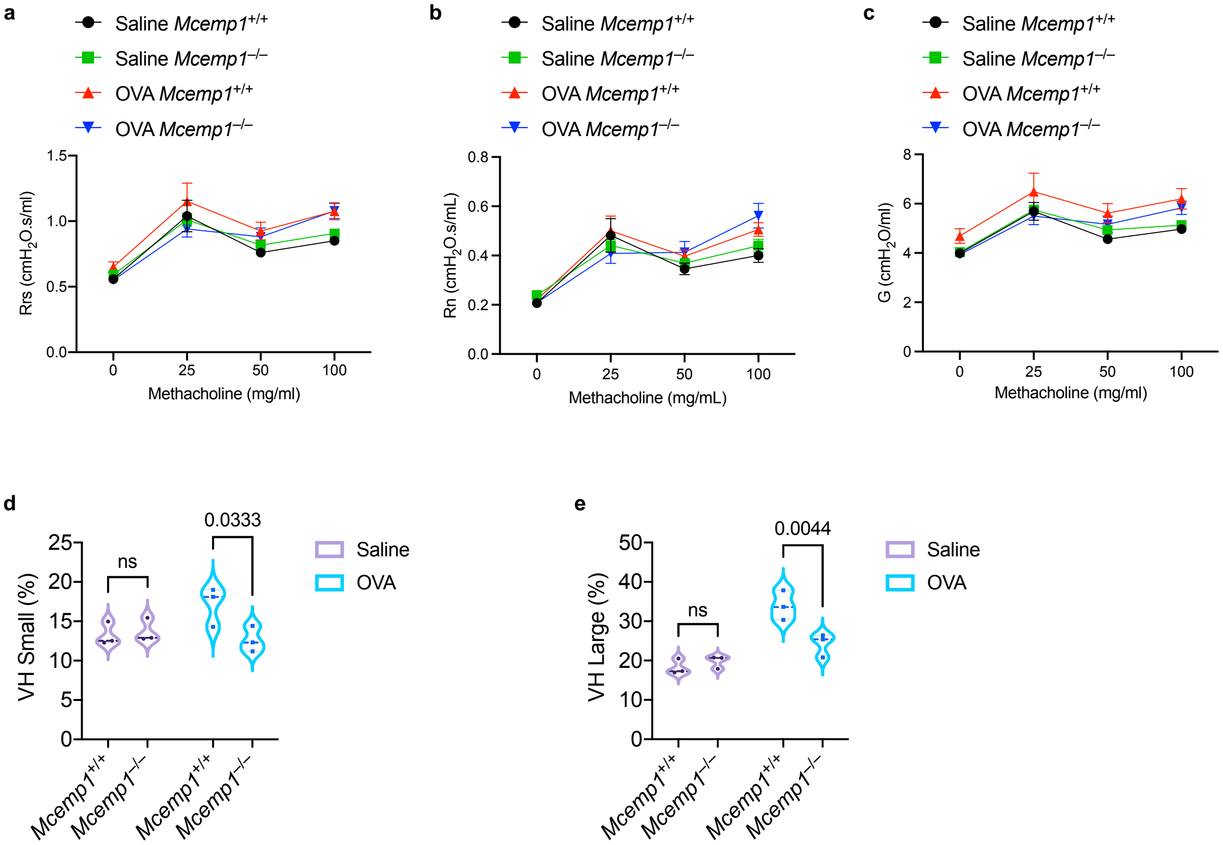

Supplementary Figure 7 **The effect of MCEMP1 deficiency on lung resistance and lung ventilation.**

a,**b**,**c** Lung function parameters, resistance of respiratory system (Rrs), newtonian resistance (Rn, central airway), and tissue damping (G, alveoli) in response to the indicated concentrations of methacholine were measured 1 day after the eighth intranasal challenge with OVA or saline (n=3 for saline group, n=4 for OVA group). **d**,**e**, Ventilation metrics; ventilation heterogeneity (VH) of small or large scale (n=3 per group). Data are presented as mean ± s.d. in **a**,**b**,**c** and as violin plots with lines at median and quartiles in **d**,**e**. *P*-values were determined by two-way ANOVA with Sidak's multiple comparison in **d**,**e**. ns, not significant.

Fig. 1b

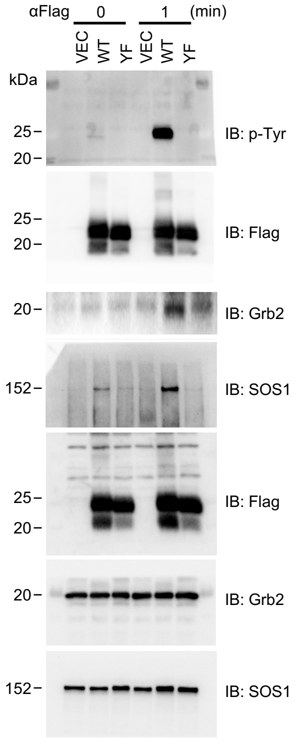

Fig. 1c

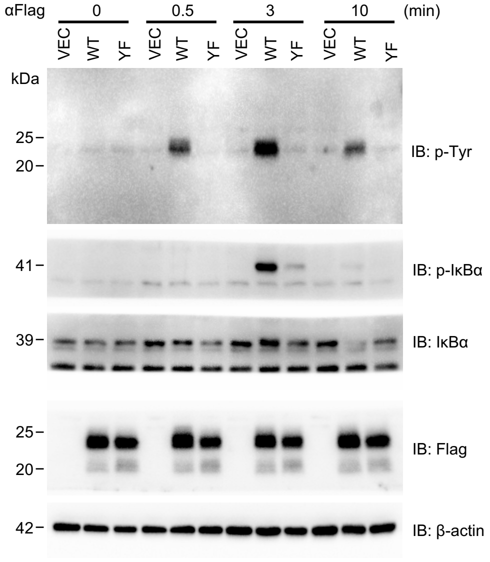

Fig. 1d

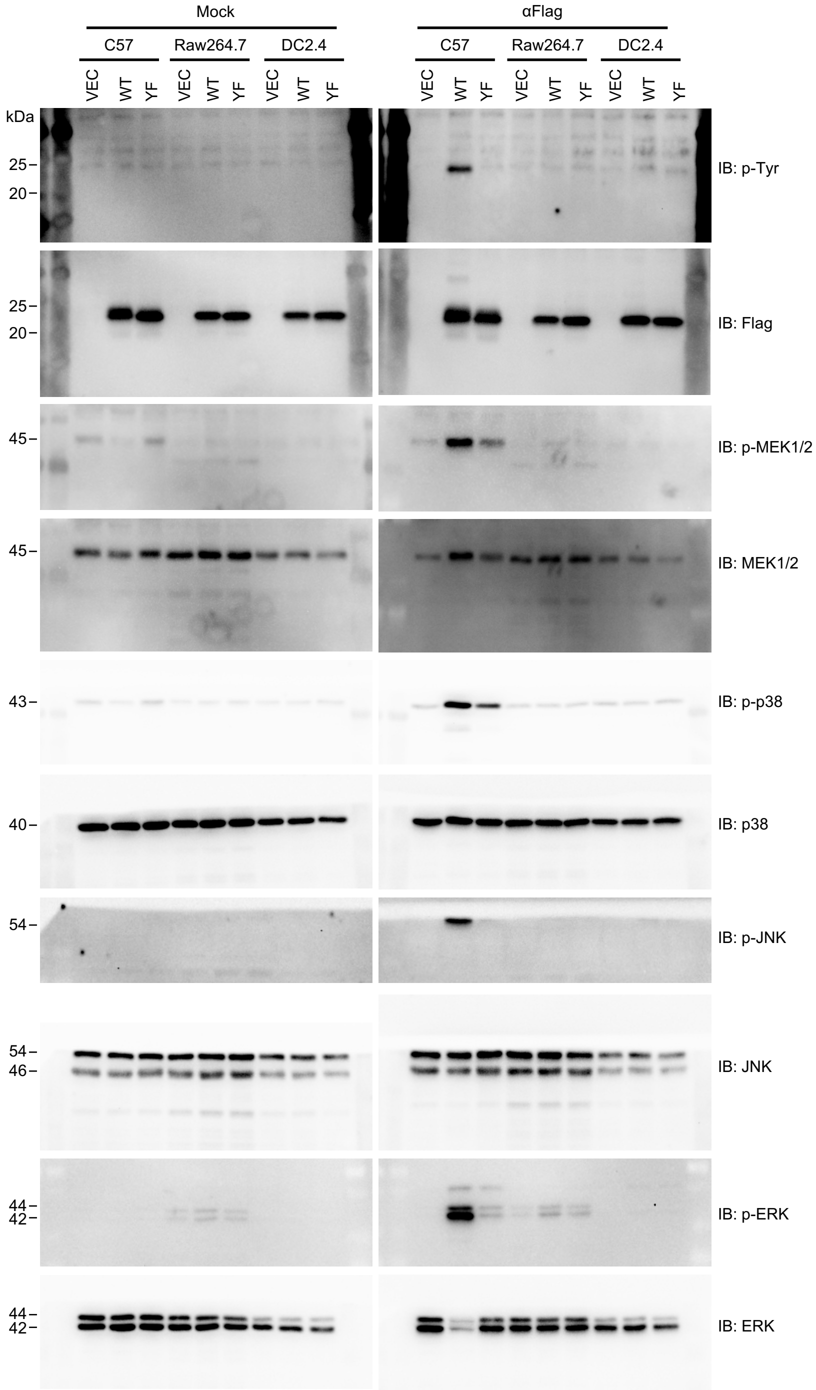

Fig. 2a

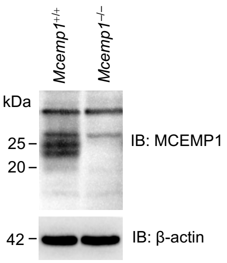

Fig. 3a

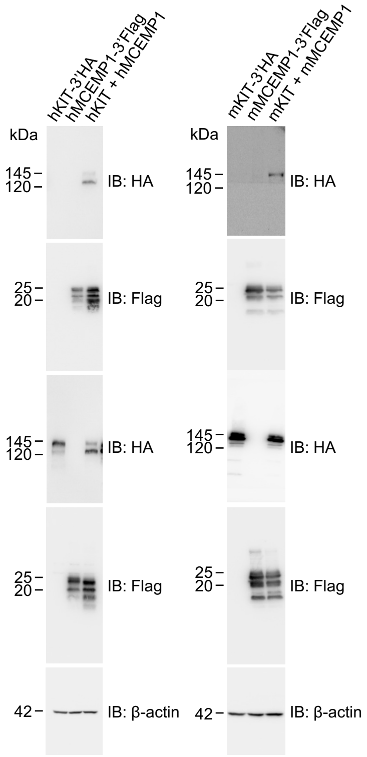

Fig. 3b

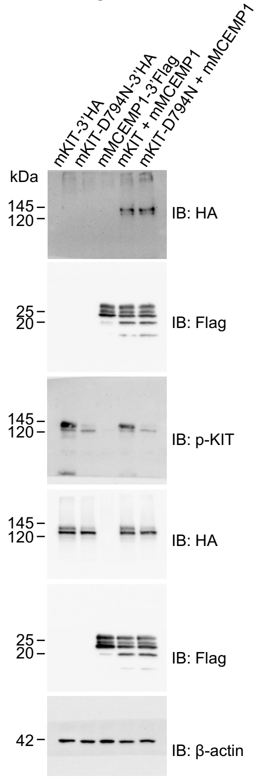

Fig. 3c

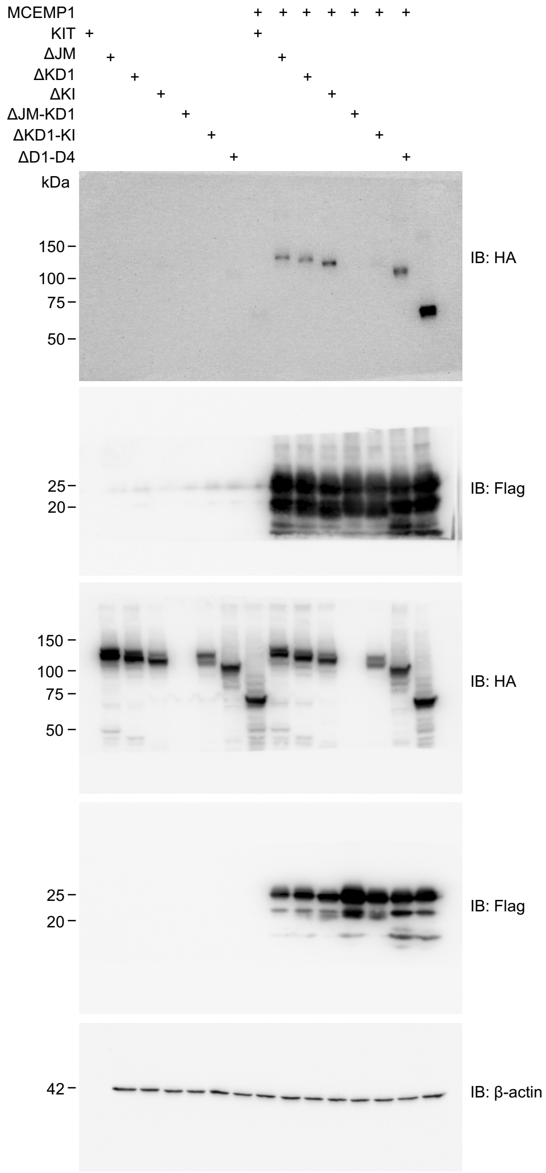

Fig. 3d

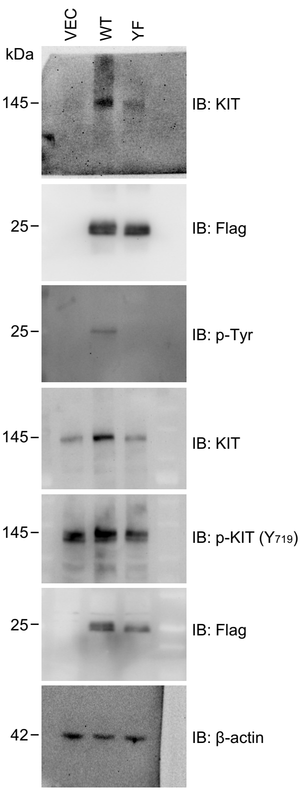

Fig. 3e

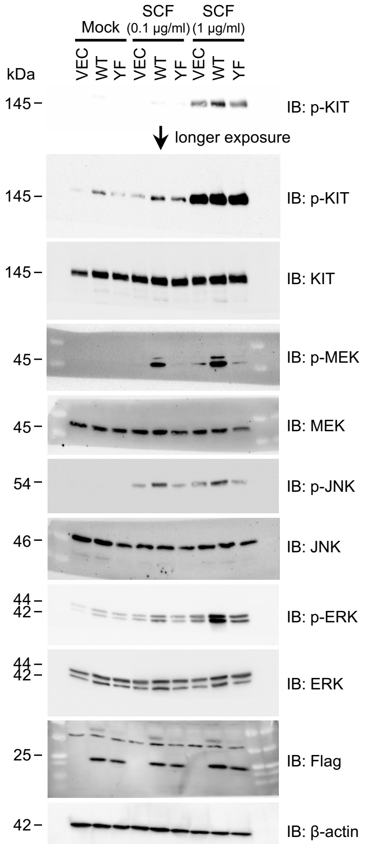

SFig. 1b

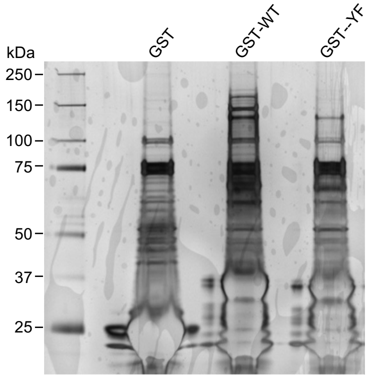

SFig. 1c

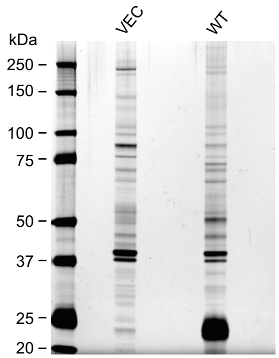

SFig. 1d

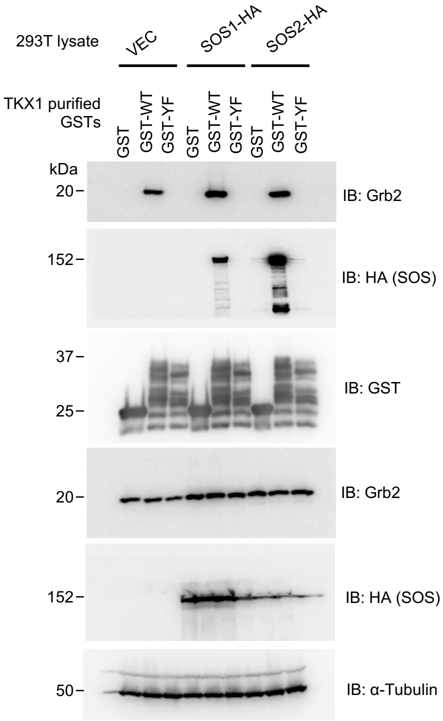

SFig. 1e

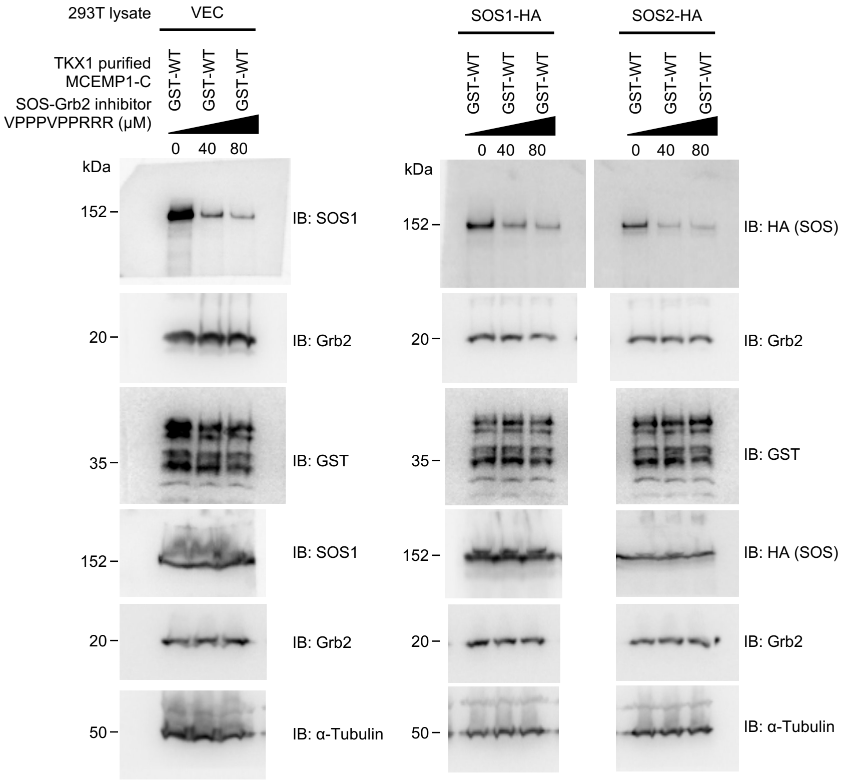

SFig. 1f

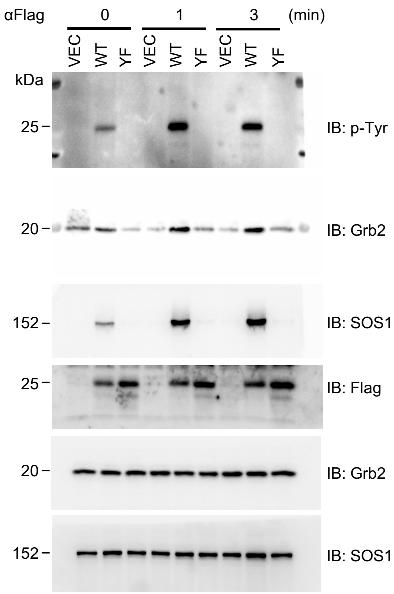

SFig. 3a

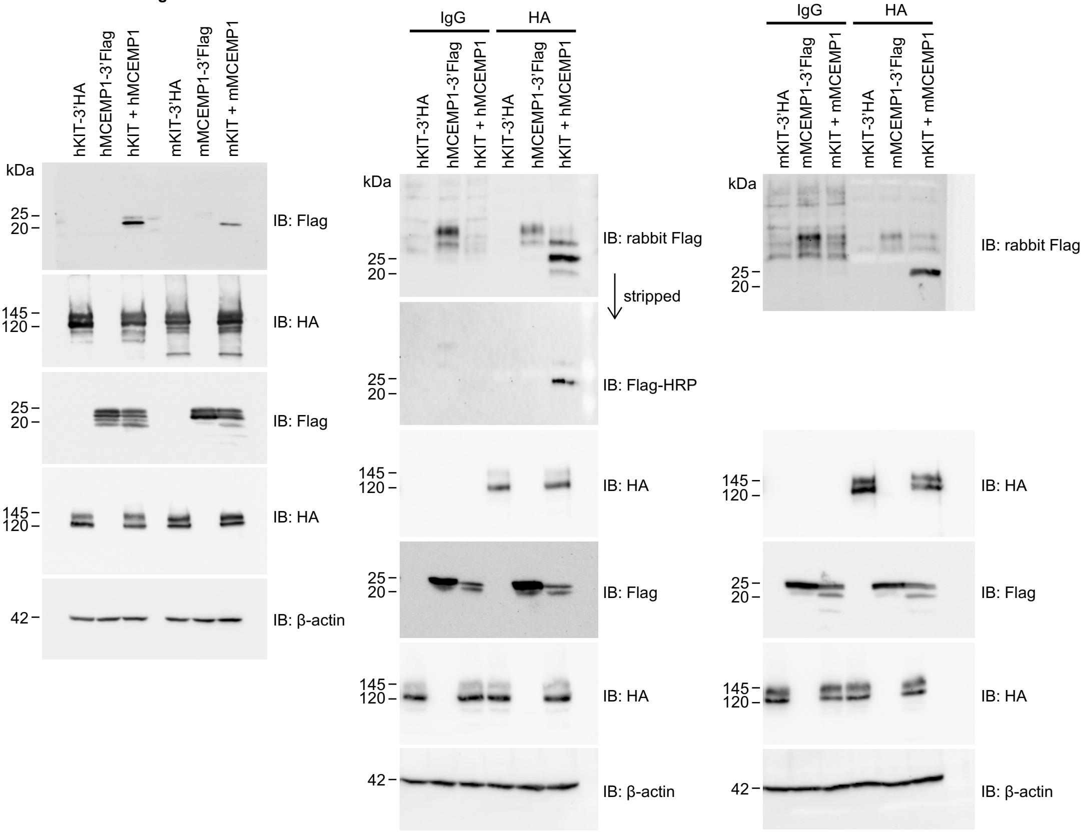

SFig. 3b

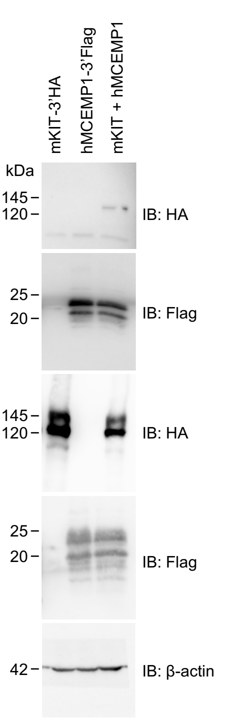

SFig. 3c

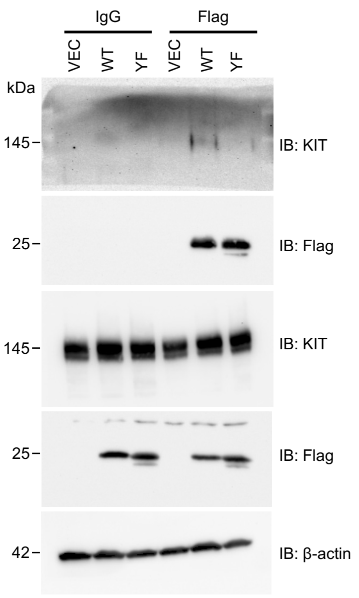

SFig. 3d

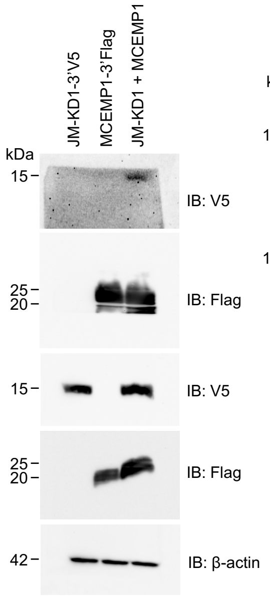

SFig. 3e

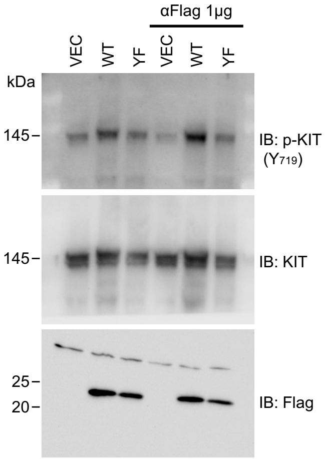

SFig. 3f

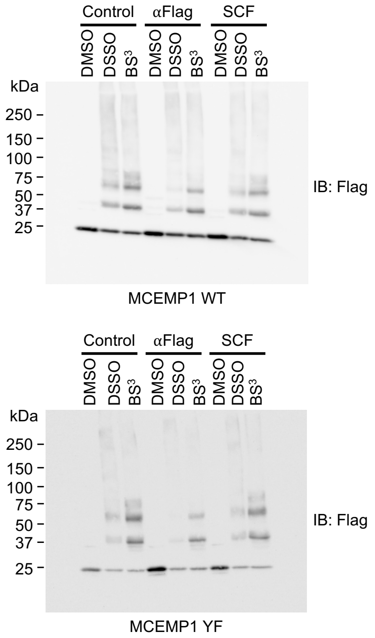

Supplement: Supplementary file 1 — Supplementary Information [file 41467_2023_37873_MOESM1_ESM.pdf]
