# Supplementary material for: Lung-specific MCEMP1 functions as an adaptor for KIT to promote SCF-mediated mast cell proliferation
Source: Nat Commun. 2023 Apr 11;14:2045. doi: 10.1038/s41467-023-37873-3 (PMC10090139; doi:10.1038/s41467-023-37873-3)
Supplement: Supplementary file 2 — Reporting Summary [file 41467_2023_37873_MOESM2_ESM.pdf]

nature portfolio

Corresponding author(s): Youn Jung Choi, Jae U. Jung

Last updated by author(s): Mar 13, 2023

Reporting Summary

Nature Portfolio wishes to improve the reproducibility of the work that we publish. This form provides structure for consistency and transparency in reporting. For further information on Nature Portfolio policies, see our Editorial Policies and the Editorial Policy Checklist.

Statistics

For all statistical analyses, confirm that the following items are present in the figure legend, table legend, main text, or Methods section.

n/a	Confirmed	
☐	☒	The exact sample size (*n*) for each experimental group/condition, given as a discrete number and unit of measurement
☐	☒	A statement on whether measurements were taken from distinct samples or whether the same sample was measured repeatedly
☐	☒	The statistical test(s) used AND whether they are one- or two-sided<br>*Only common tests should be described solely by name; describe more complex techniques in the Methods section.*
☐	☒	A description of all covariates tested
☐	☒	A description of any assumptions or corrections, such as tests of normality and adjustment for multiple comparisons
☐	☒	A full description of the statistical parameters including central tendency (e.g. means) or other basic estimates (e.g. regression coefficient) AND variation (e.g. standard deviation) or associated estimates of uncertainty (e.g. confidence intervals)
☐	☒	For null hypothesis testing, the test statistic (e.g. *F*, *t*, *r*) with confidence intervals, effect sizes, degrees of freedom and *P* value noted<br>*Give P values as exact values whenever suitable.*
☒	☐	For Bayesian analysis, information on the choice of priors and Markov chain Monte Carlo settings
☒	☐	For hierarchical and complex designs, identification of the appropriate level for tests and full reporting of outcomes
☒	☐	Estimates of effect sizes (e.g. Cohen's *d*, Pearson's *r*), indicating how they were calculated

Our web collection on statistics for biologists contains articles on many of the points above.

Software and code

Policy information about availability of computer code

Data collection	N/A
Data analysis	For RNA-seq, demultiplexed fastq files were analyzed with Partek Flow software, v10.0. We used R (Core Team 2014) to analyze RNA-seq data and figures were produced with the ggplot2 package (Wickham, 2009). Pathway analysis was performed using Ingenuity Pathway Analysis (QIAGEN, version 01-20-04). Flow cytometry data was analyzed using FlowJo v10.0 software. For Statistics, we used GraphPad Prism 9.0. Image J was used to measure densitometric analysis and count number of toluidine blue positive cells.

For manuscripts utilizing custom algorithms or software that are central to the research but not yet described in published literature, software must be made available to editors and reviewers. We strongly encourage code deposition in a community repository (e.g. GitHub). See the Nature Portfolio guidelines for submitting code & software for further information.

Data

Policy information about availability of data

All manuscripts must include a data availability statement. This statement should provide the following information, where applicable:

- Accession codes, unique identifiers, or web links for publicly available datasets
- A description of any restrictions on data availability
- For clinical datasets or third party data, please ensure that the statement adheres to our policy

Source data are provided with this paper. RNA-seq data generated in this study have been deposited in the GEO database under accession code GSE197042; Subseries, GSE197040 (lung) and GSE197041 (PCMC).

Human research participants

Policy information about studies involving human research participants and Sex and Gender in Research.

Reporting on sex and gender	N/A
Population characteristics	N/A
Recruitment	N/A
Ethics oversight	N/A

Note that full information on the approval of the study protocol must also be provided in the manuscript.

Field-specific reporting

Please select the one below that is the best fit for your research. If you are not sure, read the appropriate sections before making your selection.

☒ Life sciences ☐ Behavioural & social sciences ☐ Ecological, evolutionary & environmental sciences

For a reference copy of the document with all sections, see nature.com/documents/nr-reporting-summary-flat.pdf

Life sciences study design

All studies must disclose on these points even when the disclosure is negative.

Sample size	No statistical methods were used to predetermine sample size. Instead sample size was determined based on the numbers required to generate statistical power and preliminary experiments. A small pilot experiment showed biologically significant results and so a larger scale experiment was performed based on results from pilot experiment.
Data exclusions	No data were excluded from the analyses.
Replication	All experiments were repeated at least twice to ensure reproducibility of findings. Details are clearly indicated in each figure legend.
Randomization	All samples were assigned to groups randomly.
Blinding	During sample collection and processing and downstream analysis, there was no subjective component.

Reporting for specific materials, systems and methods

We require information from authors about some types of materials, experimental systems and methods used in many studies. Here, indicate whether each material, system or method listed is relevant to your study. If you are not sure if a list item applies to your research, read the appropriate section before selecting a response.

Materials & experimental systems

n/a	Involved in the study
[ ]	[x] Antibodies
[ ]	[x] Eukaryotic cell lines
[x]	[ ] Palaeontology and archaeology
[ ]	[x] Animals and other organisms
[x]	[ ] Clinical data
[x]	[ ] Dual use research of concern

Methods

n/a	Involved in the study
[x]	[ ] ChIP-seq
[ ]	[x] Flow cytometry
[x]	[ ] MRI-based neuroimaging

Antibodies

Antibodies used

Antibodies included MCEMP1 (polyclonal rabbit made by GST-mMCEMP1-ectodomain fusion protein), Phospho-KIT (Cell Signaling, 3391S), KIT (Cell Signaling, 3074S), SOS1 (Cell Signaling, 12409S), Grb2 (Santa Cruz, C-23), 4G10 (EMD Millipore, 05-321), 4G10-HRP (EMD Millipore, 16-105), P-Tyr-1000 (Cell Signaling, 8952S), Phospho-IκBα (Cell Signaling, 9241S), IκBα (Cell Signaling, 4814S), Phospho-MEK1/2 (Cell Signaling, 9154S), MEK1/2 (Cell Signaling, 9122S), Phospho-p38 (Cell Signaling, 9211S), p38 (Cell Signaling, 9212S), Phospho-JNK (Cell Signaling, 9251S), JNK (Cell Signaling, 4668S), Phospho-ERK (Cell Signaling, 4370S), ERK (Cell Signaling, 4695S or 9101S), GST (Santa Cruz, B-14), Actin (Santa Cruz, C4), Tubulin (Santa Cruz, B7), rabbit-Flag (Sigma, F7425), mouse-Flag (Sigma, F1804), rabbit-HA (Covance, PRB-101P, BioLegend 902302), mouse-HA (BioLegend 901503, 16B12), mouse Flag-HRP (abcam ab49763, M2), mouse HA-HRP (BioLegend 901519, 16B12), mouse V5-HRP (Thermo R961-25), mouse IgG-HRP (Cell Signaling, 7076), and rabbit IgG-HRP (Cell Signaling, 7074), anti-2,4,dinitrophenyl antibody (Sigma, D8406, Clone SPE-7).

Validation

All the antibodies have been validated for the species and application following information provided by the manufacturer, and titrated using appropriate positive and negative controls, using different antibody dilutions. Further information on antibodies for immunoblotting is presented in Supplementary Table 3.

Eukaryotic cell lines

Policy information about cell lines and Sex and Gender in Research

Cell line source(s)

293T, Raw264.7, HMC-1 cells were purchased from ATCC. C57 and DC2.4 cells were kindly provided by S.J. Galli and K.L. Rock, respectively.

Authentication

None of the cell lines used have been authenticated.

Mycoplasma contamination

All cell lines were tested negative for mycoplasma contamination.

Commonly misidentified lines
(See ICLAC register)

No commonly misidentified cell lines were used.

Animals and other research organisms

Policy information about studies involving animals; ARRIVE guidelines recommended for reporting animal research, and Sex and Gender in Research

Laboratory animals

Mcemp1–/– mice (C57BL/6) were generated by CRISPR-Cas9 system. B6D2F1 (C57BL/6 X DBA2) foster female mice were used. Details are reported in Methods. Mice were maintained in a barrier facility for animals in a temperature-controlled system characterized by 22 Celsius degrees and 50% humidity, with a 12 hours dark/light cycle within cages.

Wild animals

No wild animals were used in the study.

Reporting on sex

male and female mice of equal distribution were used.

Field-collected samples

No field collected samples were used in the study.

Ethics oversight

All mice were bred and housed in specific pathogen free facilities maintained by the USC Animal Research Center at Keck Medical School and Cleveland Clinic Lerner Research Institute. All animal experiments were reviewed and approved by Institutional Animal Care and Use committees (IACUC) and Institutional Biosafety Committee (IBC) at USC and Cleveland Clinic.

Note that full information on the approval of the study protocol must also be provided in the manuscript.

Flow Cytometry

Plots

Confirm that:

☒ The axis labels state the marker and fluorochrome used (e.g. CD4-FITC).

☒ The axis scales are clearly visible. Include numbers along axes only for bottom left plot of group (a 'group' is an analysis of identical markers).

☒ All plots are contour plots with outliers or pseudocolor plots.

☒ A numerical value for number of cells or percentage (with statistics) is provided.

Methodology

Sample preparation	Lungs were mechanically dispersed using scissors and enzymatically digested using 1 ml of 1.67 mg/ml collagenase IV (Worthington, LS004210) plus 10 µl of 10 mg/ml DNase Type IV (Sigma, D5025) per lobe for 1 h at 37 °C with shaking. After filtering through 40 µm strainer and RBC lysis (BioLegend, 420301), cells were incubated with Fc block in 50 µl FACS buffer for 10 min at 4 °C, then incubated with antibody cocktail of interest in total 100 µl FACS buffer for 30 min at 4 °C, washed with FACS buffer, and fixed with 4% paraformaldehyde for 15 min at 4 °C. Information on antibodies for flow cytometry is provided in Supplementary Table 6.
Instrument	FACS acquisition was performed on BD FACS Celesta (BD Biosciences) and Sony ID7000 (Sony).
Software	All flow cytometry data were analyzed using FlowJo v10.0 software.
Cell population abundance	In all experiments, negative control (non-staining), single color controls, and Fluorescence Minus One (FMO) controls, and/or a control sample lacking primary antibody were included. The abundance of cell populations is reported as either a percentage of the parent population or an absolute number which is calculated using cell counting bead.
Gating strategy	FSC-A/SSC-A (cells) -> FSC-H/FSC-A (single cells) -> Blue Dead Cell Stain negative (live cells) -> Representative gating plots are presented in each figure.

☒ Tick this box to confirm that a figure exemplifying the gating strategy is provided in the Supplementary Information.
