# Supplementary figures and images for: Lung-specific MCEMP1 functions as an adaptor for KIT to promote SCF-mediated mast cell proliferation
Source: Nat Commun. 2023 Apr 11;14:2045. doi: 10.1038/s41467-023-37873-3 (PMC10090139; doi:10.1038/s41467-023-37873-3)

Fig. 1b

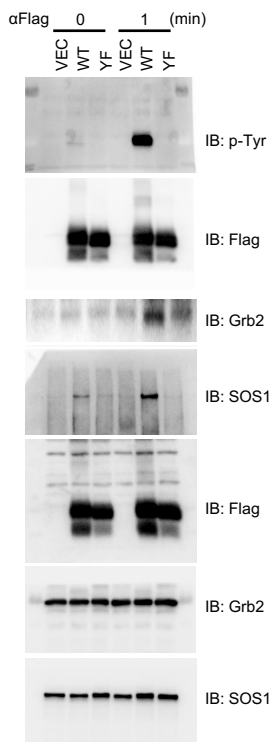

Fig. 1c

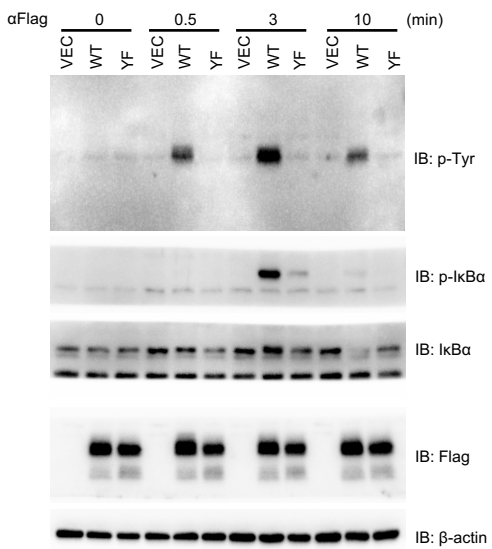

Fig. 1d

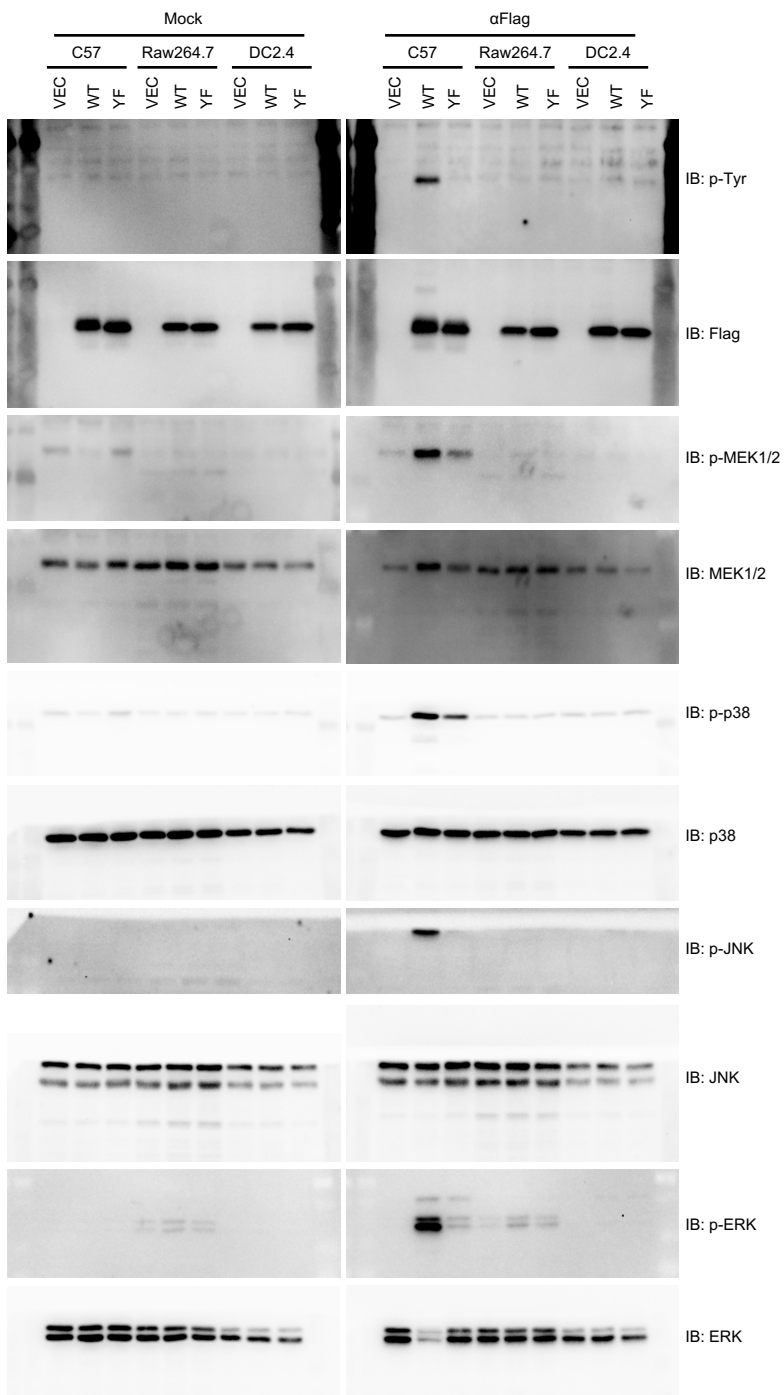

**Fig. 2a**

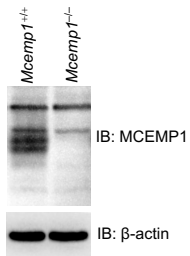

**Fig. 3a**

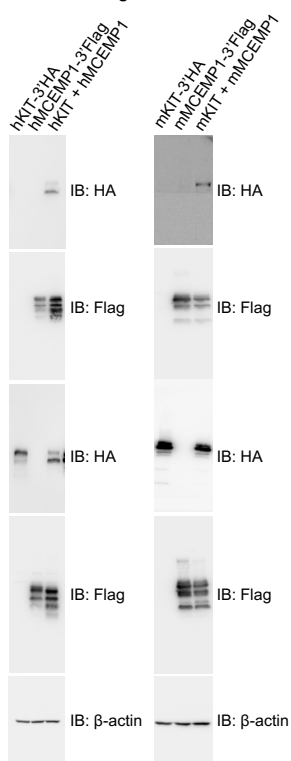

**Fig. 3b**

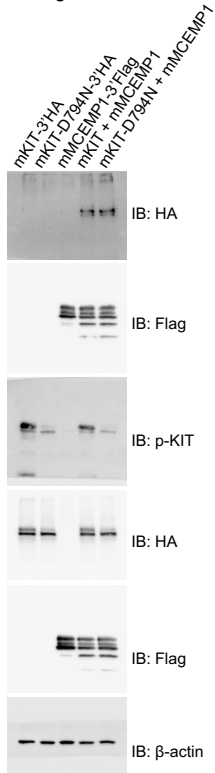

**Fig. 3c**

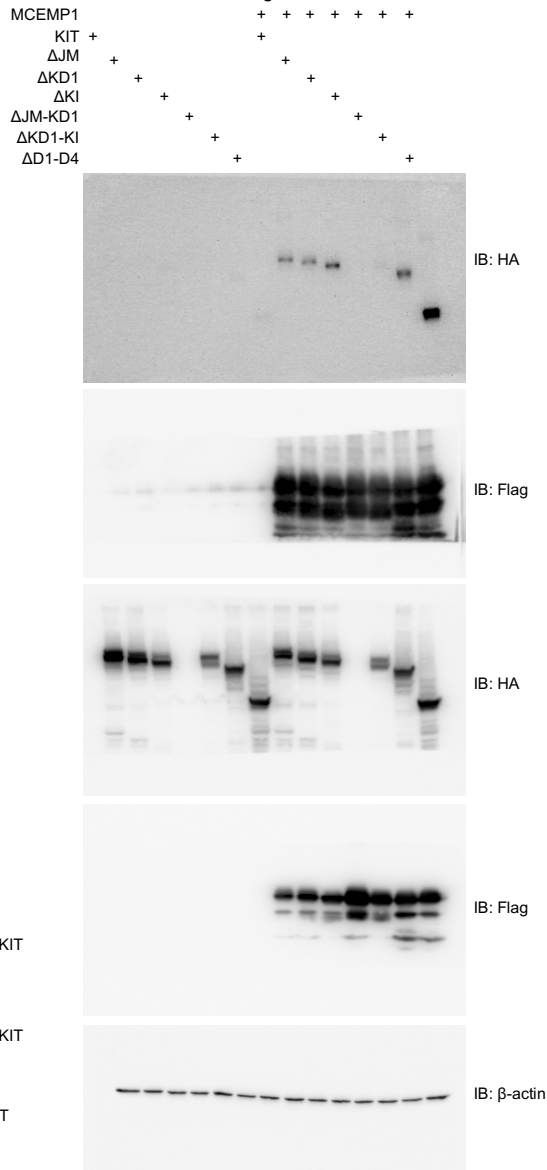

**Fig. 3d**

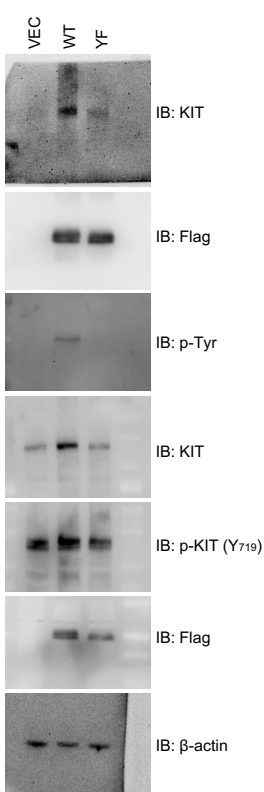

**Fig. 3e**

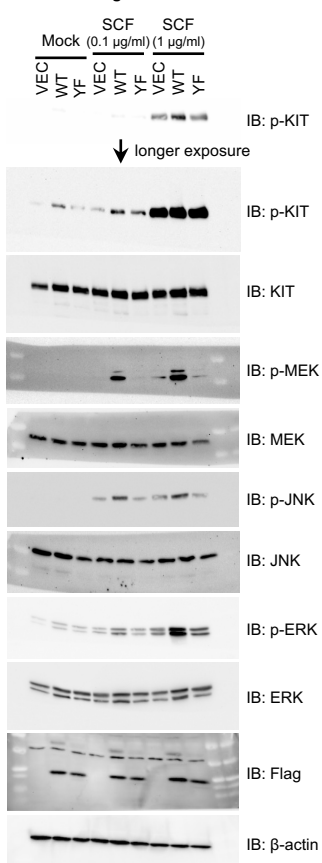

SFig. 1b

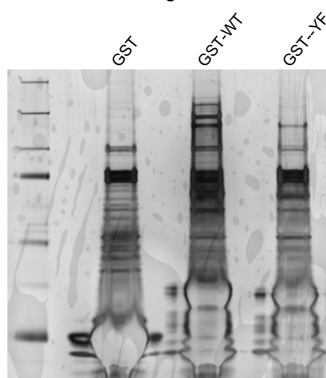

SFig. 1c

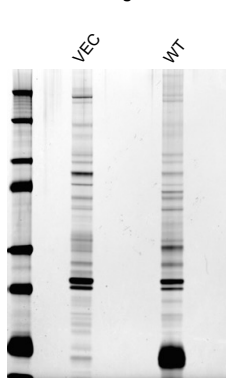

SFig. 1d

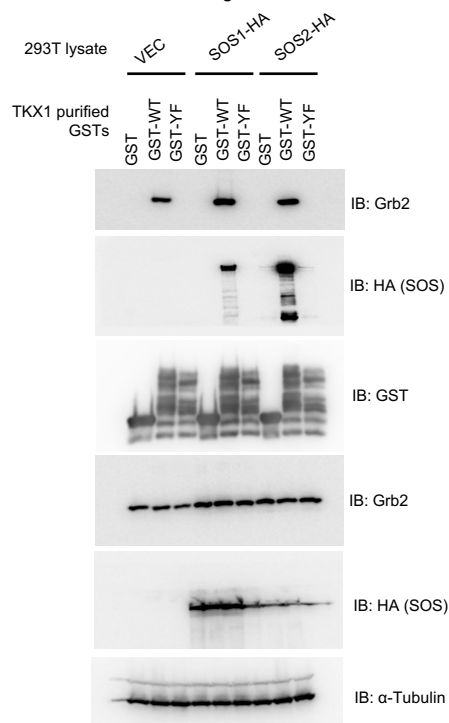

SFig. 1e

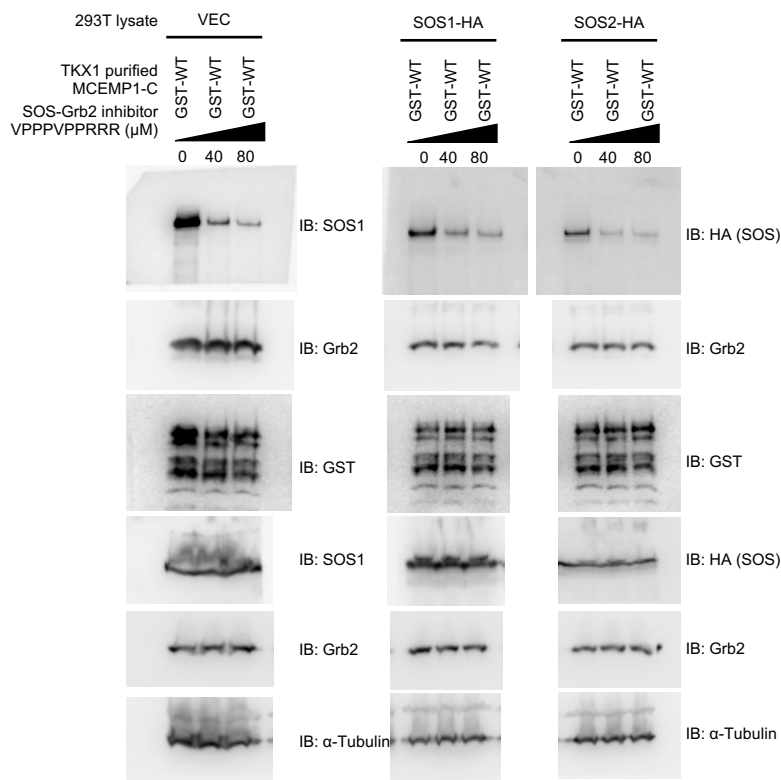

SFig. 1f

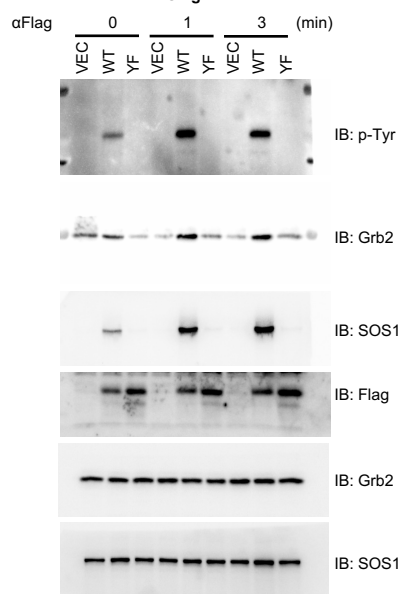

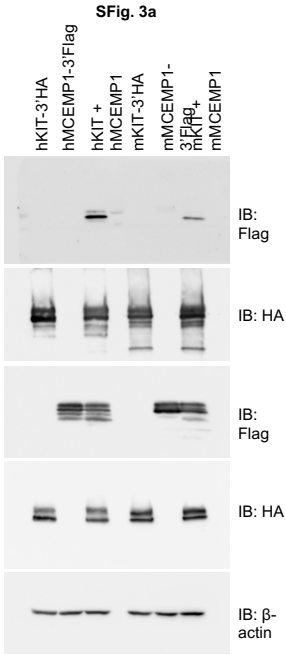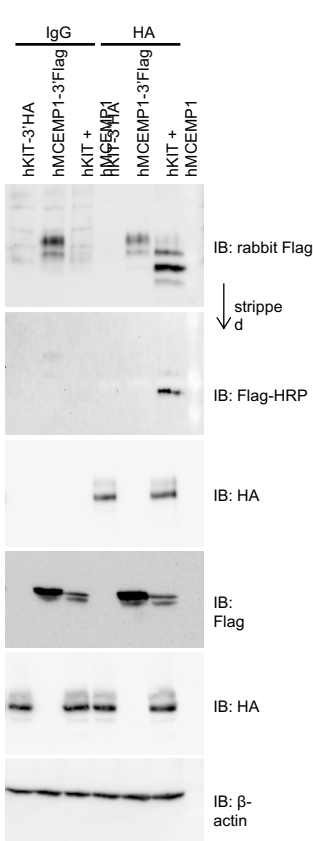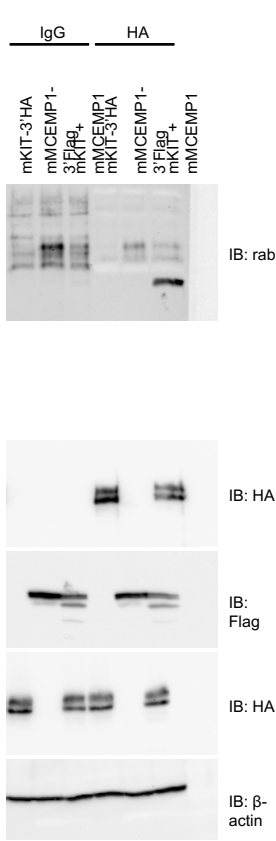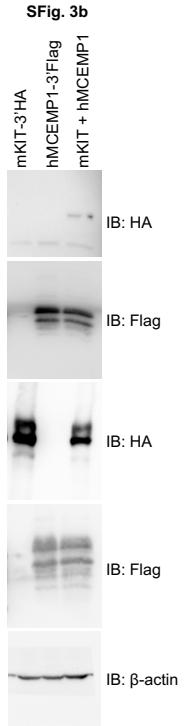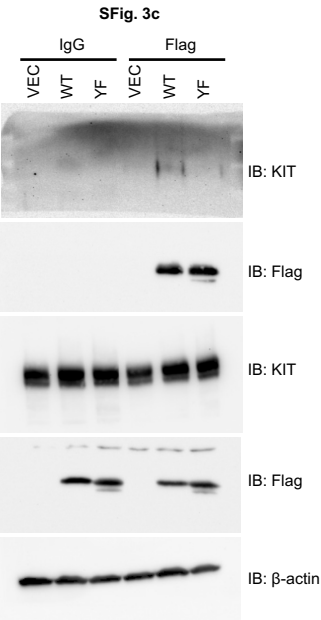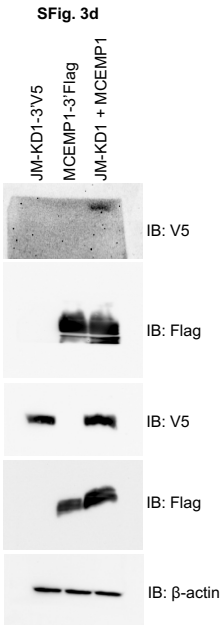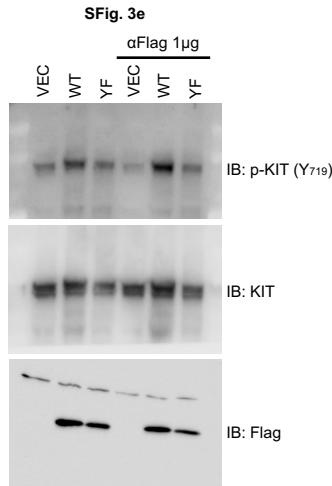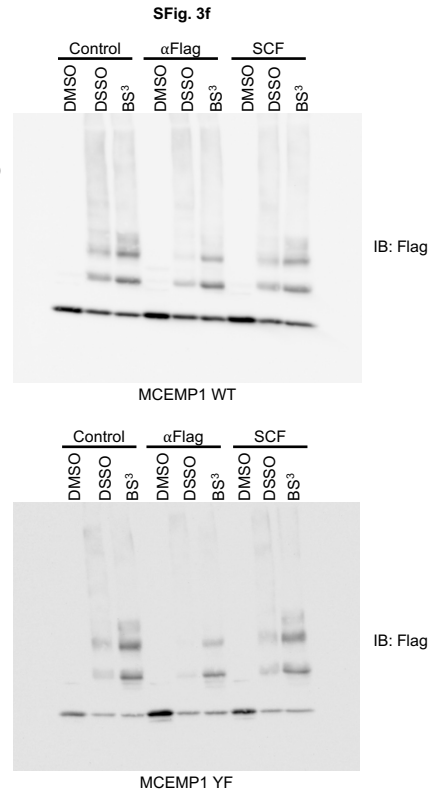

Supplement: Supplementary file 3 — Source Data [file 41467_2023_37873_MOESM3_ESM.zip › Source Data_Uncropped images.pdf]
